# A high temporal/spatial resolution neuro-architecture study of rodent brain by wideband echo planar imaging

**DOI:** 10.1038/s41598-021-98132-3

**Published:** 2021-10-12

**Authors:** Po-Wei Cheng, Tzi-Dar Chiueh, Jyh-Horng Chen

**Affiliations:** 1 Graduate Institute of Biomedical Electronic and Bioinformatics, National Taiwan University, Taipei, Taiwan; 2 Department of Electrical Engineering, National Taiwan University, Taipei, Taiwan; 3 Department of Electrical Engineering, Interdisciplinary MRI/MRS Lab, National Taiwan University, Taipei, Taiwan

**Keywords:** Diffusion tensor imaging, Functional magnetic resonance imaging, Magnetic resonance imaging

## Abstract

Latest simultaneous multi-slice (SMS) methods greatly benefit MR efficiency for recent studies using parallel imaging technique. However, these methods are limited by the requirement of array coils. The proposed Coherent Wideband method, which employs an extended field of view to separate multiple excited slices, can be applied to any existing MRI instrument, even those without array coils. In this study, the Coherent Wideband echo-planar imaging method was implemented on 7 T animal MRI to exhibit comprehensive enhancements in neuro-architecture, including diffusion tensor imaging (DTI) and functional MR studies (fMRI). Under the same scan time, the time-saving effect can be manipulated to increase the number of averages for DTI SNR improvement, reducing fractional anisotropy difference by 56.9% (from 0.072 to 0.041) and the deviation angle by 64% (from 25.3° to 16.2°). In summary, Coherent Wideband Echo Planar Imaging (EPI) will provide faster, higher resolution, thinner slice, or higher SNR imaging for precision neuro-architecture studies.

## Introduction

Magnetic resonance imaging (MRI)^1^ technology is a non-invasive diagnostic tool that reveals not only changes in cerebral blood oxygen saturation^2,3^ but also pathways in nerve fibers^4–6^. However, compared with computed tomography and positron emission tomography, MRI has a markedly lower time resolution.

Consequently, numerous acceleration techniques were born in pursuit of greater MRI throughput. Simultaneous multi-slice (SMS) imaging is an acceleration technique which enhances imaging efficiency by simultaneously exciting and acquiring multiple slices. Müller first proposed the concept in 1988 and used the Fourier shift theorem to develop multi-frequency selective radiofrequency pulses for SMS^7^. This led to the proposal of Hadamard-encoded RF pulses, which Souza et al. utilized to separate simultaneously acquired slices^8^. Glover et al. took a similar approach, utilizing encoded RF pulses in their phase-offset multi-planar (POMP) technique^9^. These earliest SMS methods didn’t shorten MR scan time due to the extra phase-encoding steps or excitations needed in their design. However, they facilitated the development of future SMS techniques in the next few decades.

Modern SMS methods can be divided into two categories according to their hardware dependency. The majority of SMS methods belong to the first category, which utilizes additional hardware to provide necessary spatial information to separate simultaneously acquired slices. Multi-channel coils were first proposed and used by Larkman to acquire multiple excitations^10^. Afterward, Breuer used a combination of RF and multi-channel coils for multi-slice imaging^11^. Furthermore, Setsompop et al. combined controlled aliasing in parallel imaging with echo-planar imaging (EPI) to match multi-excited image signals with standard signals^12,13^. These SMS techniques hugely advanced the research in fields of functional MRI (fMRI) and diffusion tensor imaging (DTI)^14,15^. Numerous studies successfully proved the benefits of applying SMS techniques instead of other techniques, such as increasing analysis bandwidth^16^ or enhancing image resolution^17^.

However, all aforementioned SMS methods suffer from hardware-induced artifact problems like g-factors and slice leakage due to the reconstruction of undersampling^18^. Since small animal pre-clinical apparatuses generally possess fewer coils, the noise will be even more severe. From this aspect, non-hardware based SMS approaches are essential to provide neural dynamics in preclinical studies.

Weaver et al*.* first proposed the approach to achieve simultaneous multi-slice imaging acceleration without extra hardware in 1988^19^. The multi-excited signals can be separated by applying an extra gradient during spatial encoding, but this addition results in severe image blur. Therefore, Wu et al*.* developed the Multi-frequency excited Wideband (ME-Wideband)^20^ method on gradient sequences for blur mitigation, improving Wideband image quality to the standard level. Since most current SMS techniques use additional hardware, non-hardware based SMS techniques are more commonly referred to as “Wideband” techniques to emphasize the difference^21,22^.

For dynamic functional studies, Nunes's work first applied gradient blips to EPI in a "Blipped-wideband" manner^23^. Setsompop et al. applied Weaver’s technique to EPI and referred to it^12^. Naturally, Blipped-wideband suffers from the same blurring problem. For blur mitigation, the ME-wideband technique is applied to EPI in this paper. We adapt ME-wideband to EPI sequence, the “Coherent Wideband” technique is proposed to optimize wideband EPI images.

Coherent Wideband, consisting of novel refocusing gradient sequence and precise phase alignment, provides faster, higher resolution, thinner slice imaging, or higher signal-to-noise pre-clinical imaging for precision neuro-architecture studies. Experimental results prove the advantages of Coherent Wideband against previous Wideband methods and demonstrate the benefits of this technique in fields of pre-clinical DTI and fMRI researches.

## Materials and methods

### Pulse sequence and imaging parameters

This paper proposes the “Coherent Wideband” technique, consisting of a novel refocusing gradient sequence and precise phase alignment. This study introduced a method that employs expanded field of view (FOV) to acquire image signals from simultaneously-excited slices without extra hardware, then utilizes separation gradients so that image signals undergo phase shifts. Although additionally applied separation gradient and expanded FOV can separate image signal from different slices on the image domain, they also cause residual artifacts or “N/2 ghosting” artifacts. To eliminate these artifacts, an accurate phase alignment method is introduced by this study.

#### Coherent wideband EPI sequence

For EPI sampling, extra separation gradient pulses were applied along the z-direction to achieve phase shift in signals and corresponding image separation. For two-fold excitation (acceleration factor, *W* = *2*), each *k*-line exhibits an extra phase of pi/2, which results in a FOV/2 shift on the image.

According to the ME-wideband equation by Wu, signal strength and phase vary by this separation gradient applied as described in Eqs. (1) and (2).

1 $${S}^{^{\prime}}\left({k}_{x},{k}_{y}\right)=S\left({k}_{x},{k}_{y}\right)\times \left({\int }_{{z}_{1}}^{{z}_{2}}{\exp}\left(i\gamma {G}_{z}\tau \right)dz\right)$$

2 $$\left({\int }_{{z}_{1}}^{{z}_{2}}{\exp}\left(i\gamma {G}_{z}\tau \right)dz\right)=({z}_{2}-{z}_{1})\times sinc(\gamma {G}_{z}\tau (\frac{{z}_{2}-{z}_{1}}{2}))\times {\exp}\left(i\gamma {G}_{z}\tau (\frac{{z}_{2}+{z}_{1}}{2})\right)$$

A gradient is required to separate two adjacent planes, which further reduces the signal strength in wideband MRI. Blipped-CAIPI method^24^ and ME-Wideband^20^ have suggested that refocusing gradients can be applied to recover signal strength.

**Figure 1:**
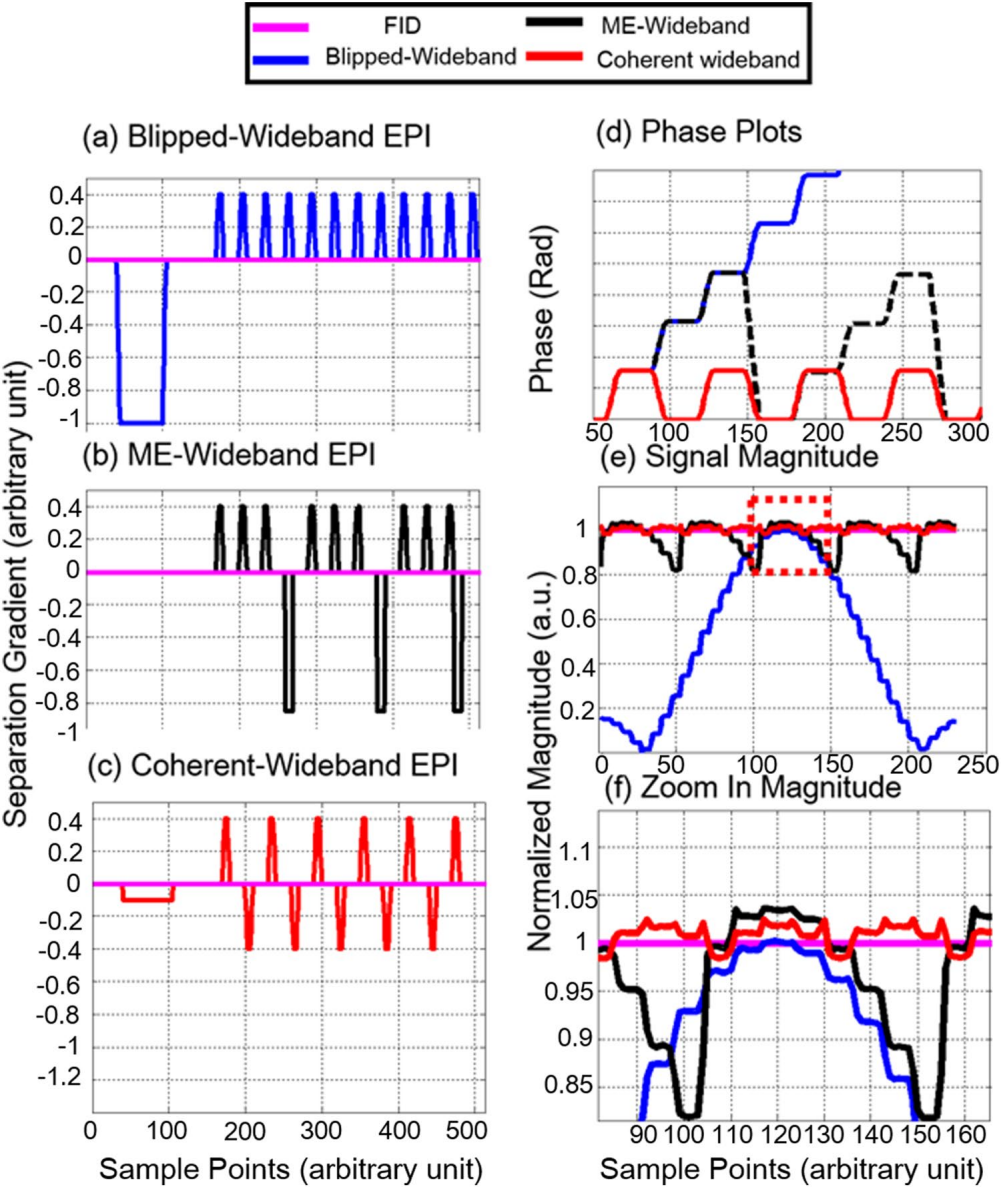
Pulse sequences of multiple wideband EPI. (**a**) Blipped Wideband EPI Sequence with constant separation gradients. (**b**) ME Wideband EPI Sequence with refocusing gradient. (**c**) Coherent Wideband EPI sequence with bipolar refocusing gradient. (**d**) Phase plots of multiple EPI sequences. (**e**) Signal Magnitude of various EPI sequences. (**f**) Zoom in View of Signal Magnitude.

Figure 1 shows multiple wideband EPI sequence designs, including blipped wideband EPI, ME-wideband EPI, and Coherent Wideband EPI. ME-wideband EPI is the EPI version of ME-Wideband, while Coherent Wideband EPI is its optimization. In blipped wideband EPI, accumulated separation gradient causes severe signal attenuation. While ME-wideband EPI partially mitigates such attenuation, the rapidly switching refocusing gradient leads to additional eddy current artifact. The optimized Coherent Wideband design keeps the isochromatic spin in phase in each pair of separation gradients, minimizing signal attenuation, phase deviation, and eddy current artifacts (see Fig. 1d–f). The phase plots of Coherent Wideband show the phase shifts between 0 and pi/2 which causes an N/2 shift on the image domain. The following section will demonstrate how an accurate phase alignment process addresses this issue.

#### Zero phase N/2 artifact mitigation: EPI reconstruction

**Figure 2:**
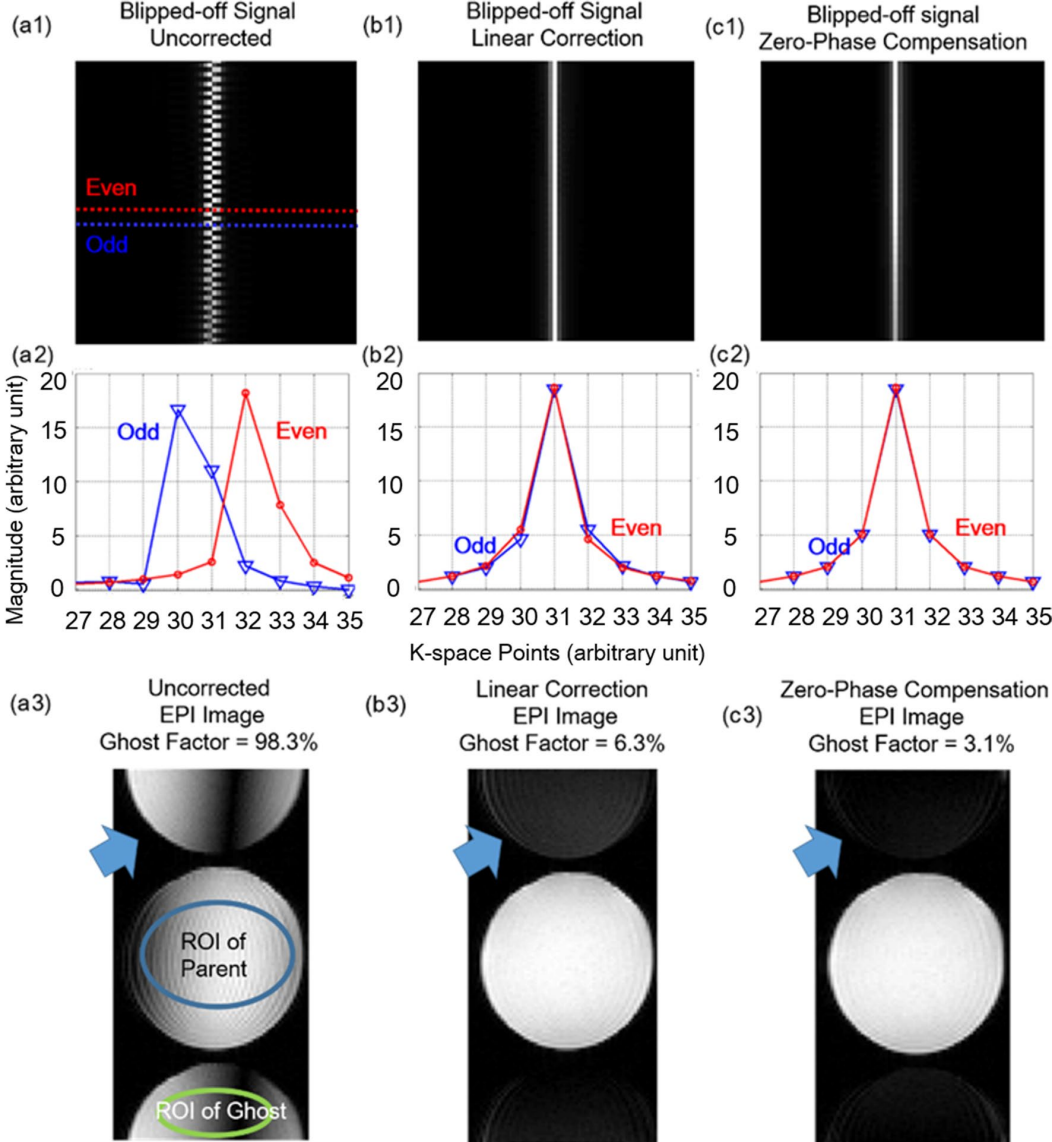
EPI Phase Alignment. Multiple N/2 ghost correction method is implemented in (**a**), (**b**) and (**c**). The uncorrected N/2 ghost artifact is shown in (**a.3**). The artifact is partially removed by the linear correction method in (**b.3**), and thoroughly removed by the zero-phase compensation method in (**c.3**).

EPI images suffer from N/2 artifacts due to rapidly switching gradients and resulting odd–even echo phase errors. Since these artifacts overlap with multi-slice images in Wideband EPI applications, N/2 ghost removal is the priority of our image reconstruction. Proper phase corrections can be done to reduce N/2 ghost by measuring the gradient data set in Fig. 2a and b ^25^. However, ghost artifacts are still visible due to k-space signal asymmetric. The zero-phase compensation method based on Ordidge et al*.*^26^ improves the phase adjustment process, facilitating the symmetricity of k-space signals and reducing the ghost factor from 6.3 to 3.1% (Fig. 2c). Nevertheless, for the case of wideband EPI, the phase difference caused by additional separation gradient should also be considered. The next section will elaborate on this extra phase alignment process.

#### Slice dependent phase alignment: wideband reconstruction

**Figure 3:**
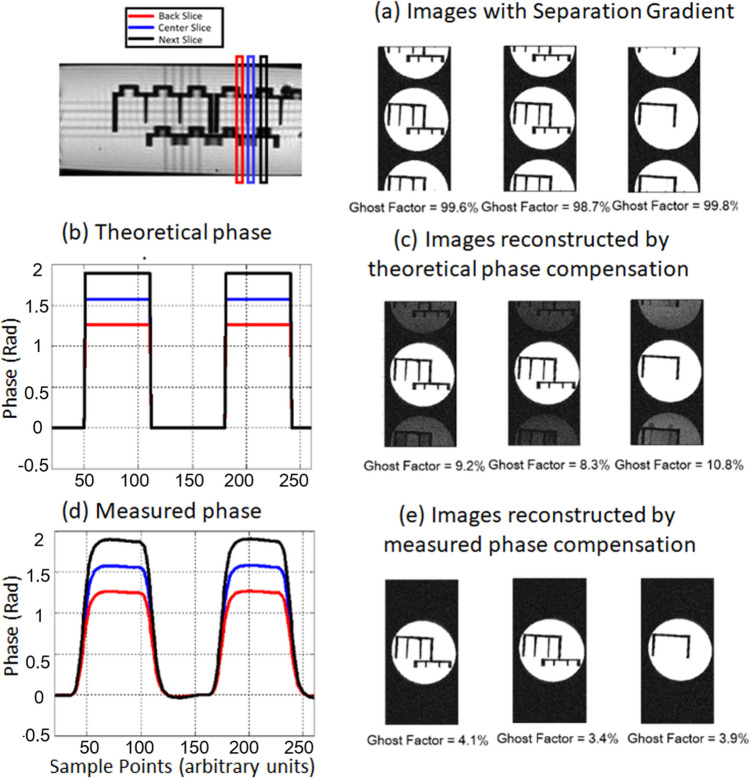
Slice Dependent Phase Alignment. Separation gradients induce phase shift in k-space which leads to N/2 artifacts on the images shown in (**a**). The artifacts were partially corrected by theoretical phase as shown in (**b**) and (**c**). By applying the measured phase, precise offset compensation for Wideband images was ensured as shown in (**d**) and (**e**).

The Wideband technique utilizes additional separation gradients to separate simultaneously-excited images. These gradients introduce different amounts of phase to signals depending on the spatial location of the excited slices, which also contributes to N/2 artifacts as shown in Fig. 3(a). Therefore, this extra phase must be measured and compensated to ensure image quality. Ideally, this phase (Fig. 3b) can be calculated with the given sequence design, and phase correction can be done accordingly to remove ghost artifacts. However, the actual phase value is affected by the inhomogeneity of magnetic field and eddy currents, so remnant artifacts could still be observed after phase correction with theoretical phase values, as shown in Fig. 3(c). To ensure precise phase correction for Wideband image reconstruction, the gradient measurement method proposed by Beaumont^27^ is applied in this study to determine the magnitude and phase of signals, as shown in Fig. 3(d). As Fig. 3(e) demonstrates, the ghost artifacts are no longer visible after phase correction with measured phase values.

#### Similarity and difference between blipped-CAIPI and coherent wideband EPI

There are three main differences between Coherent-Wideband EPI and Blipped-CAIPI EPI:

1. Coherent-Wideband EPI can be implemented on any MRI without multi-channel coils.
2. Coherent-Wideband EPI uses increased FOV to achieve SMS instead of multi-channel coils so that image reconstruction will not be affected by g-factor.
3. Coherent-Wideband EPI acquires more sampling points to effectively keep the signal-to-noise ratio.

Both Coherent-Wideband EPI and Blipped-CAIPI adopted bipolar gradients, but the gradient strength for the two techniques is different, which results in different signal shifts in the image domain. For Blipped-CAIPI, the simultaneously acquired slices shift by FOV/2 so the images will overlap. Blipped-CAIPI solves the overlap issue with multi-coil sensitivity information, but the reconstructed images suffer from g-factor and leakage problems caused by ill-conditioned under-sampling reconstruction; On the other hand, Coherent-wideband EPI increases phase-encoding lines to accommodate the simultaneously acquired slices, therefore the images will not overlap.

#### Animals

In this study, pre-clinical scans were performed on the male healthy Sprague Dawley rat model (200-300 g, N = 6 in this study) from National Taiwan University Laboratory Animal Center. Pre-clinical experimental procedures were approved by the Institutional Animal Care and Use Committee of National Taiwan University. All animal experimentation was done with institutional approval and followed NTU guidelines. This study is conducted in accordance to ARRIVE guidelines.

#### MR parameters

All experimental data used in this study was obtained on the Bruker 7 T MRI system by a volume coil (Biospec 70/30, PV5.1, Bruker BioSpin MRI GmbH, Ettlingen, Germany). All experimental protocols were approved by Use Committee of National Taiwan University.

- *Rodent brain structure study: High temporal resolution*
  Coherence Wideband EPI achieves twofold acceleration for rodent brain scan. The scan parameters are as follows: For conventional Spin-Echo EPI, TR/TE = 3000/75 ms, standard matrix size = 96 × 96, wideband matrix size = 96 × 192, slice thickness = 1 mm, resolution = 0.26 × 0.26 mm^2^, bandwidth = 250 kHz, the number of averages = 120, and the total time is 6 min. For twofold acceleration Coherent Wideband EPI, TR / TE = 1500 /75 ms, cutting the scan time to 3 min.
- *Rodent brain structure study: High spatial resolution*
  Another usage of Coherent Wideband EPI is enhancing image spatial resolution under the same scan duration. The scan parameters are as follows: For traditional Spin-Echo EPI, TR/TE = 1000/75 ms, matrix size = **80** × **80**, slice thickness = 1 mm, resolution = 0.31 × 0.31 mm^2^, bandwidth = 250 kHz, the number of averages = 400, and the total time is 6 m 40 s. For 2X-spatial resolution Coherent Wideband EPI, matrix size = **160** × **160**, TR/TE = 500/75, and the resulting scan duration is also 6 min 40 s.
- *Rodent diffusion Tensor image: High temporal resolution*
  The parameters for EPI-based Spin-Echo DTI are as follows: Standard FOV = 2.5 × 2.5 cm^2^, Wideband FOV = 2.5 × 5.0 cm^2^, slice thickness = 1 mm, standard matrix Size = 96 × 96, wideband matrix size = 96 × 192, TE = 75 ms, bandwidth = 300 kHz, echo spacing = 320us, slice number = 6, and the number of averages = 40. TR = 5000 ms for standard EPI and 2500 ms for Coherent Wideband EPI, so the respective scan time is 43 min 20 s and 21 min 40 s.
- *Rodent diffusion Tensor image: SNR enhancement*
  The time-saving advantage of the Coherent Wideband technique can also elevate image SNR by doubling the number of average for precision medicine study. In this study, the Spin-Echo DTI scan consists of 1 null-image and 12-direction diffusion gradients. The scan parameters are as follows: Standard FOV = 2.5 × 2.5 cm^2^, Wideband FOV = 2.5 × 5.0 cm^2^, slice thickness = 1 mm, TE = 75 ms, Δ/δ = 8/3 ms, b-value = 1500 s/mm^2^, bandwidth = 300 kHz, echo spacing = 320us. TR = 5000 ms for standard EPI and 2500 ms for Coherent Wideband EPI. Since Coherent Wideband EPI is two times faster, the number of average is increased from 40 to 80 while total scan time remains constant at 43 min 20 s.
- *Rodent diffusion Tensor image: Thinner Slice*
  For higher Z-axis resolution, Coherent Wideband EPI provides thinner slice imaging to delineate finer details of brain neuro-architecture. The parameters are as follows: For conventional EPI, slice thickness = 1 mm, bandwidth = 300 kHz, echo spacing = 320us, average number = 30, number of slices = 12 and TR = 5000; for Coherent Wideband EPI, slice thickness = 0.5 mm, number of average = 30, number of slices = 24 and TR = 5000. The duration of both scans is 32 m 30 s.
- *Rodent resting functional MRI: 1/2 short TR*

High sampling MR speed is one of the unmet demands for resting fMRI researches (N = 6). For investigating the quick dynamic change in fMRI, wideband EPI is utilized to improve temporal resolution in each measurement. The TR of Coherent wideband is shortened from 2 to 1 s, and the sampling point for each fMRI study is increased from **120 to 240** for better statistical significance of functional analysis under the same scan time. The alternative is to shorten the acquisition time for **120** points from 6 to 3 min to fit the user’s need.

### MR analysis

#### Diffusion analysis

The k-space data of the wideband images were reconstructed in Matlab (Math Works, Natick, USA). The DSI Studio (http://dsi-studio.labsolver.org) was used to perform Diffusion Tensor analysis and calculating the eigenvalues for each pixel. After selecting different ROIs, their respective Fractional Anisotropy (FA), Mean Diffusivity (MD), and other values can be calculated to determine their directivity and diffusivity^28,29^, and then eigenvectors can be used to calculate the direction and angle of the pixel^30^.

#### Reproducibility study

Even with the same scan parameters and conditions, different repetitions of the same DTI scan may derive different FA and fiber orientation due to thermal and systematic noise^30^ Therefore, high SNR is required for DTI to reduce the variance of analysis results and consistently track neuro-structures. To observe the reproducibility rise for SNR-enhanced DTI results, these DTI scans will be repeated twice to calculate their FA difference and deviation angle between repetitions.

#### Resting MRI analysis

Resting functional MR image analysis was performed using REST toolbox (http://www.restfmri.net/forum/), with SPM 12 (Wellcome Department of Cognitive Neurology, London, UK; http://www.fil.ion.ucl.ac.uk/spm). Default preprocessing pipeline was used to perform all the preprocessing steps, including slice-time correction, and then spatially smoothed. Physiological and other spurious noise were estimated and then removed together with the aforementioned motion artifacts by band-pass filtering (chosen band 0.001 Hz-1 Hz). The ROIs were defined and used as a SEED, and functional ROI connected to each Segment. For first-level analysis, the residual BOLD time course was extracted from each ROIs and Pearson’s correlation coefficients were computed between each pair of ROIs.

## Results

The following figure presents phantom images acquired by standard spin-echo EPI and various Wideband-EPI (W = 2) sequences in Fig. 1. For image quality evaluation, two repeat measurements were performed for each set of images to calculate its SNR^31^, standard deviation (SD) value of different interest regions^32^, and SSIM^33^. SSIM is used for measuring the similarity between two images, and the formula is based on three comparison measurements between the samples of luminance, contrast and structure. The standard EPI phantom images were used as the benchmark.

**Figure 4:**
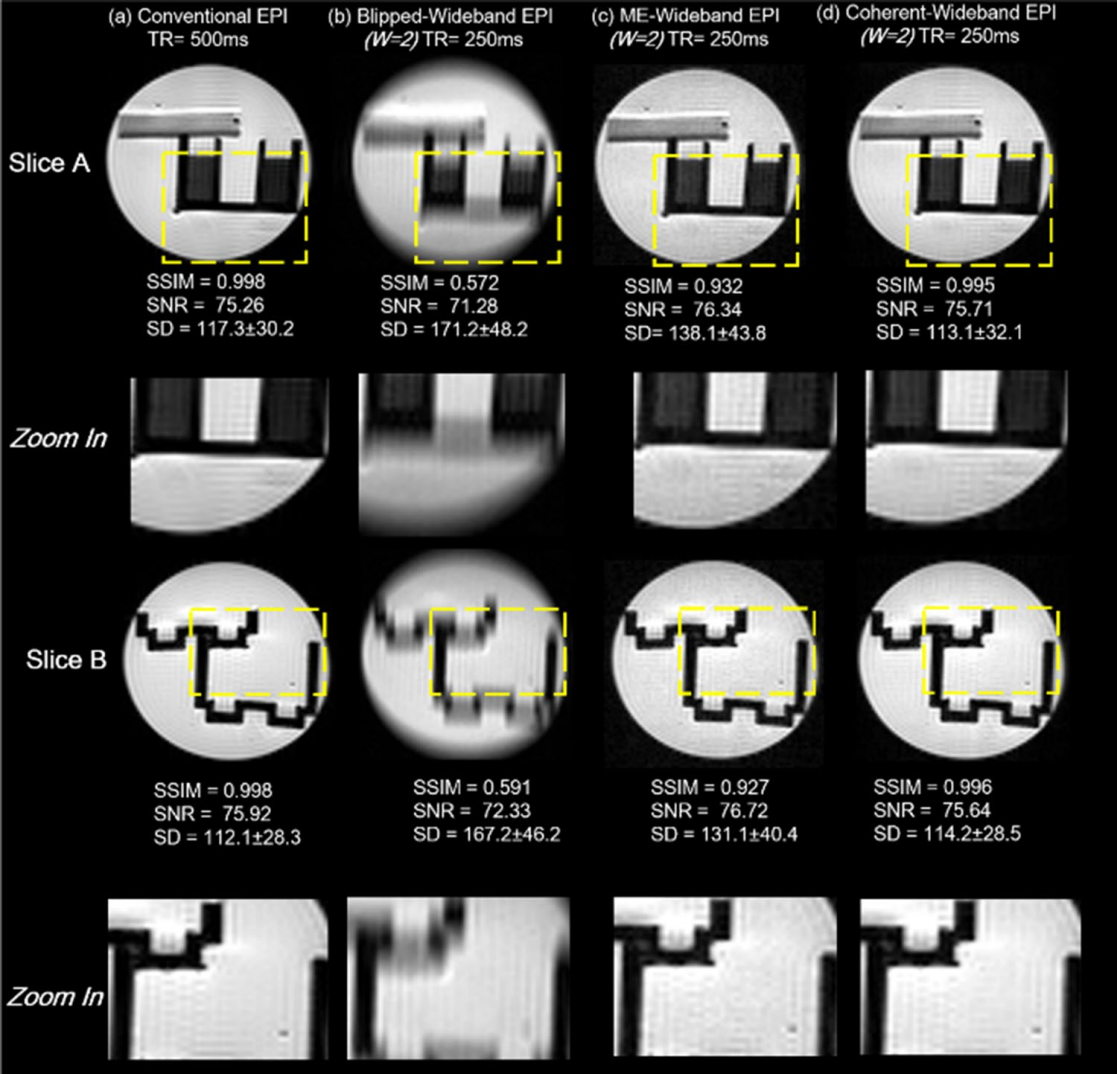
Phantom results for multiple Wideband EPI methods. (**a**) Standard EPI results (TR/TE = 500/55 ms). Slices A and B (10 mm from center respectively) were separately excited and acquired. (**b**) Blipped Wideband EPI (TR/TE = 250/55 ms) result. Slices A and B were simultaneously excited and acquired. (**c**) The ME-Wideband EPI partially mitigated the blur and showed visible inhomogeneity. (**d**) Coherent Wideband EPI results are consistent with conventional results.

Figure 4 indicates that the blipped wideband EPI, which uniformly exerts separation gradient, generated blurry images. The blur causes a drastic drop in SSIM, from 0.998 to about 0.58. The ME-Wideband EPI partially mitigated the blur improving SSIM to 0.93. However, the image results still show visible inhomogeneity as indicated by the high standard deviation value of different regions. By contrast, the Coherent EPI method, which applies bipolar separation gradients, resolved the inhomogeneity problem as SD reduces from 138.1 to 113.1 and SSIM further improves to 0.995. In summary, among all wideband methods, Coherent Wideband EPI images are consistent with conventional results, as the SNR, SD, and SSIM values indicate.

### 2X temporal resolution rodent brain results

**Figure 5:**
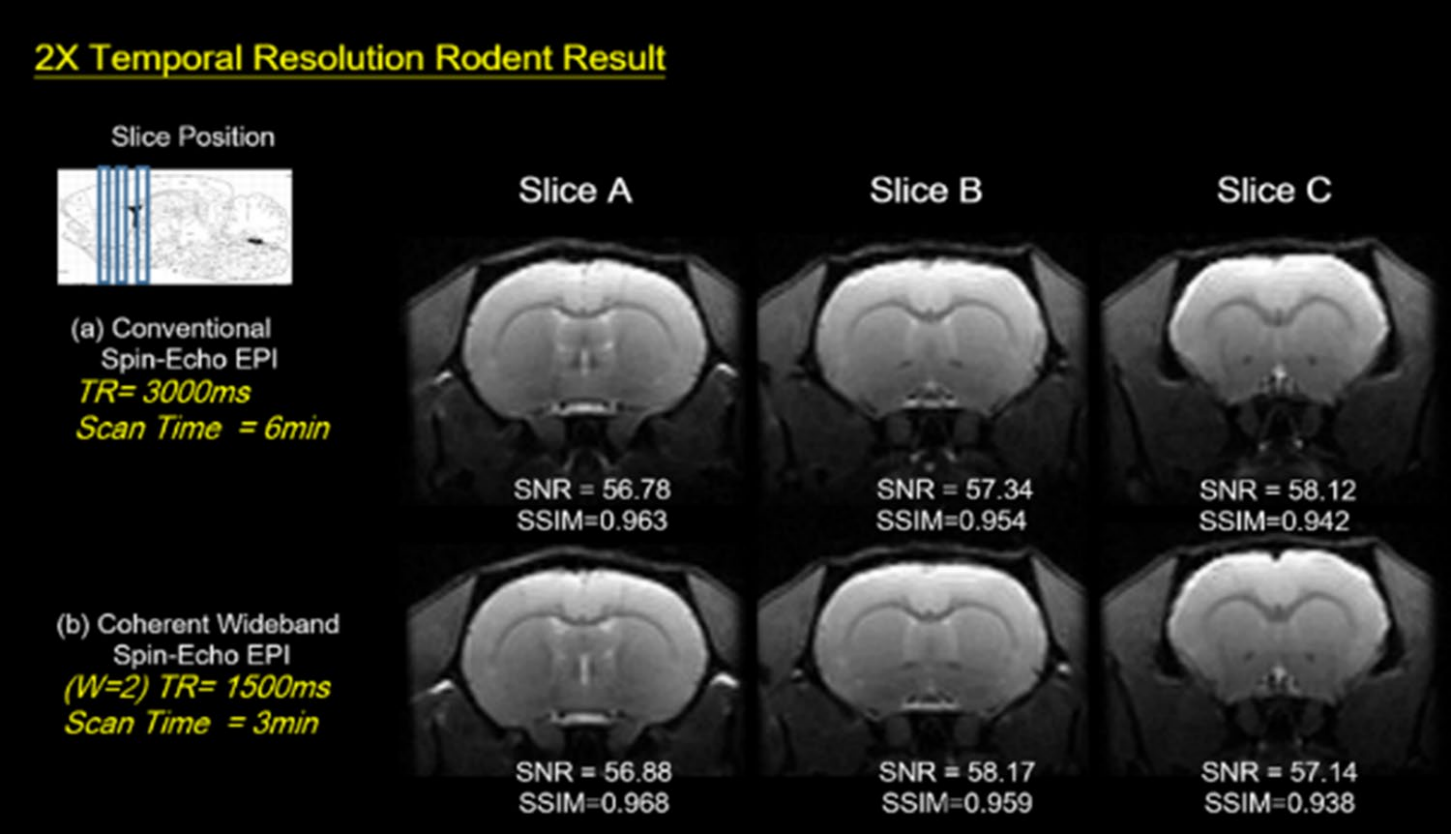
Conventional and twofold Temporal Wideband in vivo rodent brain study. The In Vivo rodent brain images were acquired by a 7 T animal MRI with (**a**) Conventional Spin-Echo EPI (**b**) Two-fold temporal resolution Coherent Spin-Echo EPI. Coherent Wideband EPI is 2 times faster while the image quality maintains the same as standard images.

Figure 5 presents the 2X Coherent Wideband spin-echo EPI in-vivo rodent brain results acquired two times faster than a conventional EPI scan, while the image quality maintains the same as standard images. The slice position is coordinated with the rodent brain atlas^34^. Quantitative comparison by SNR and SSIM values also indicate similar image quality for both methods.

### 2X spatial resolution rodent brain result

Since the Coherent Wideband technique reduces the repetition of EPI scan, Coherent Wideband EPI only needs a TR of 500 ms to perform 10 scans while conventional EPI needs 1000 ms. As the result, the number of kx and ky can be doubled for Wideband EPI to achieve higher resolution under the same scan time.

**Figure 6:**
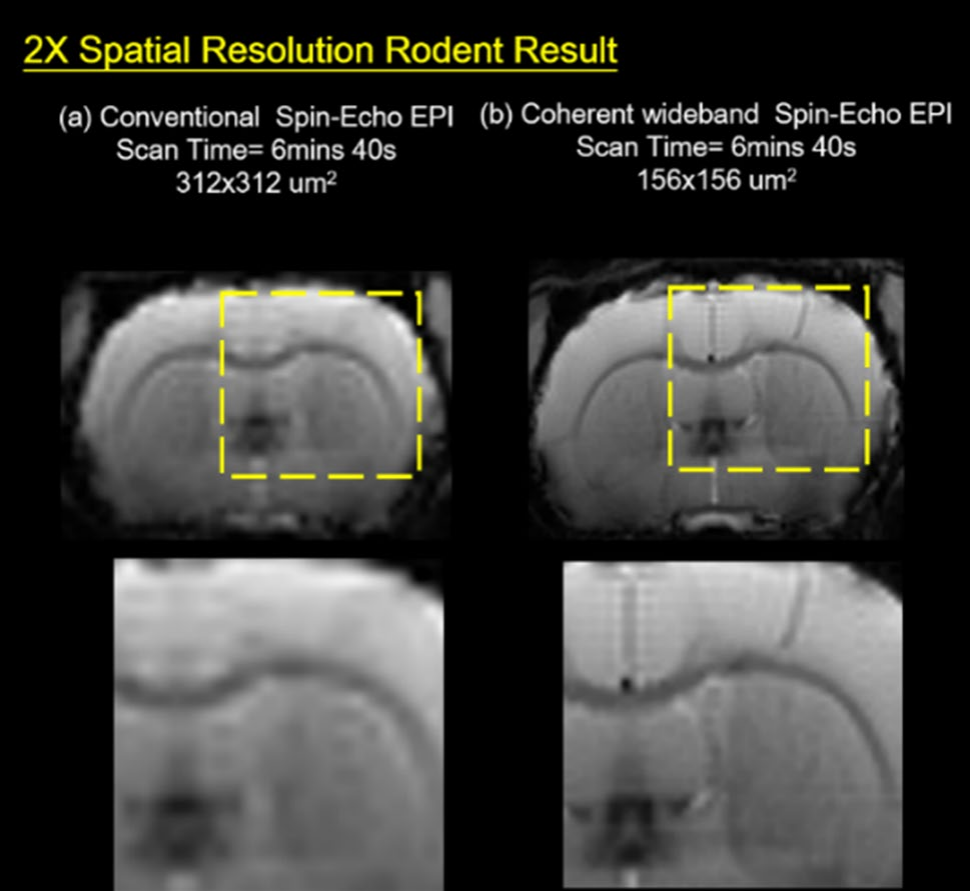
Conventional and twofold spatial resolution Wideband rodent brain results. (**a**) For standard EPI, the image resolution was 312 × 312 μm^2^ and TR = 1000 ms, so the total scan time was 6 m 40 s. (**b**) 2X Coherent Wideband acquired double data points under the same scan time, improving the image resolution to 156 × 156 μm^2^.

The spatial resolution difference between conventional and wideband EPI scans can be compared. Under the same scan time, the matrix size is 80 × 80 points for standard EPI scan and 160 × 160 for Wideband (as shown in Fig. 6).

Since the partial volume effect is reduced due to higher resolution, corresponding structural areas like blood vessels and cortical boundaries are more visible, and the demarcation for gray and white matter is clearer. Results show that the boundaries between these structures were vague due to the partial volume effect for low-resolution images. With the Wideband technique, the boundaries were much clearer due to spatial resolution enhancement.

### DTI result

**Figure 7:**
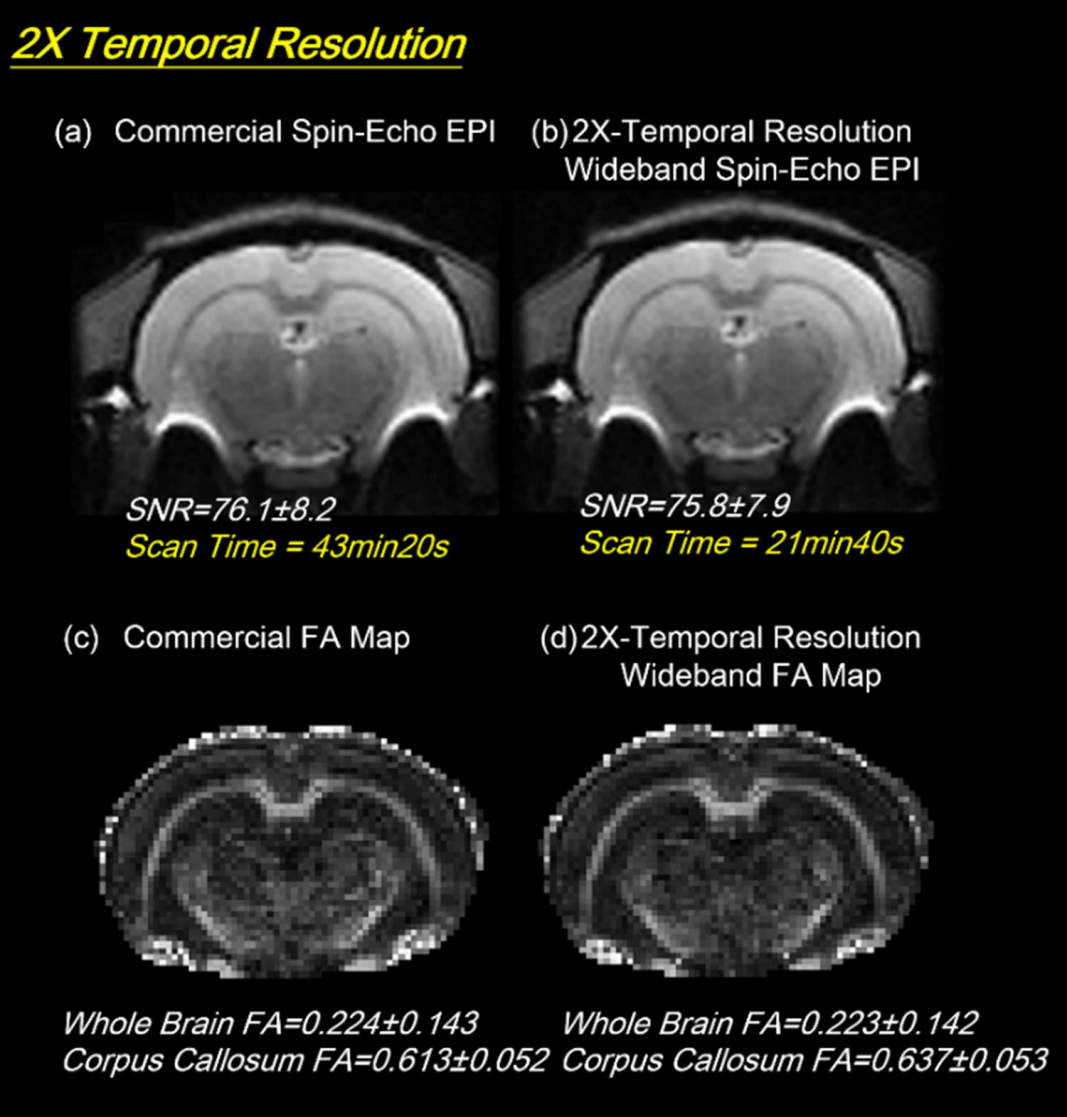
The twofold temporal resolution diffusion study. In the Coherent Wideband 2 × temporal resolution diffusion experiment, the acquisition time of the sequence is reduced from 43 min 20 s to 21 min 40 s. The signal to noise and FA value of the 2X fast image kept well at the equal quality of the original one.

**Figure 8:**
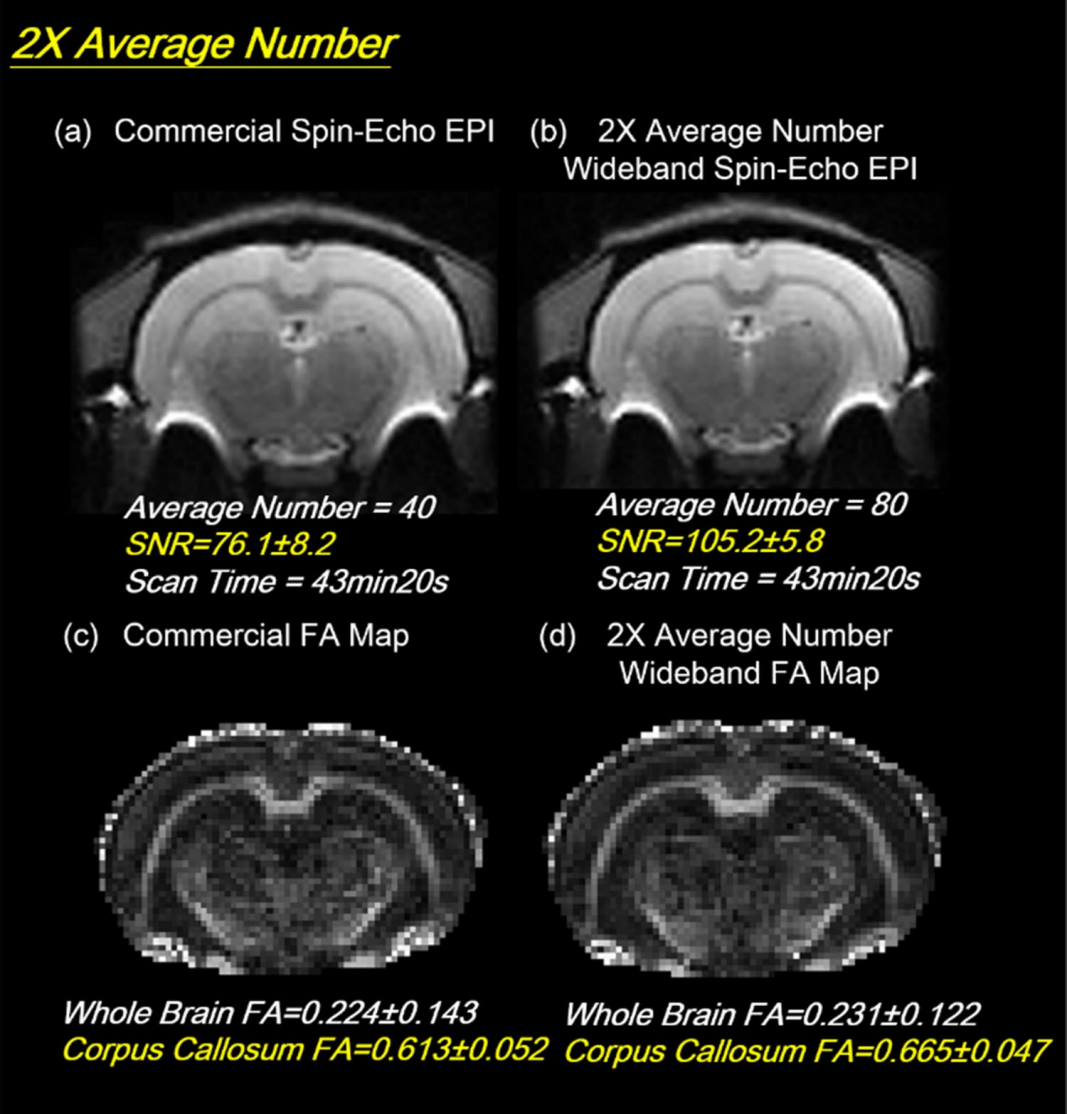
Diffusion study of 2X-Average Coherent Wideband EPI images. In the 2X-Average Coherent Wideband MR diffusion experiment, the SNR was boosted 1.4 times from 76 to 105, as expected. The acquisition time of the sequence remains at 43 min 20 s.

**Figure 9:**
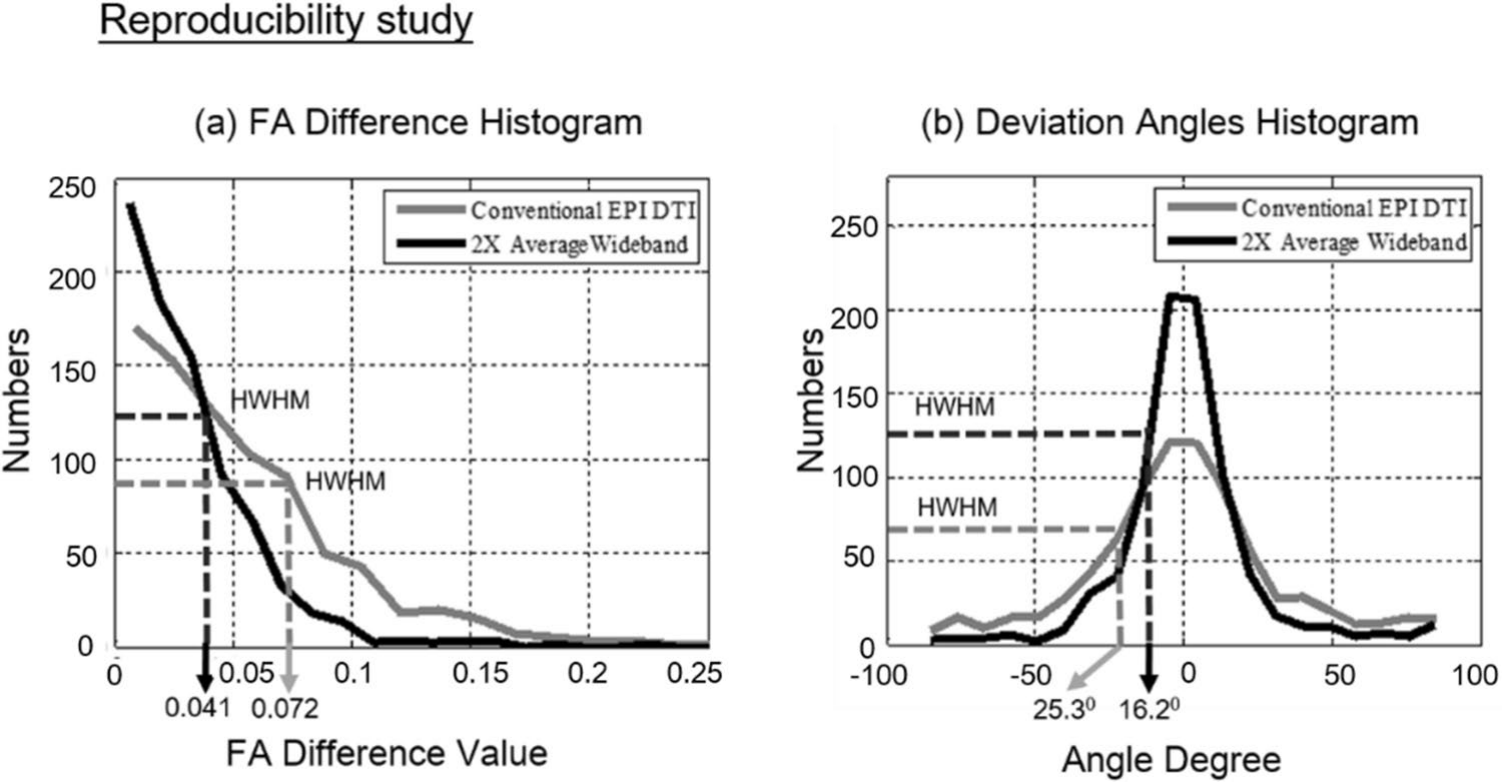
Reproducibility analysis of FA and axonal fiber orientation. The quantitative analysis of DTI results, including (**a**) fractional anisotropy (FA) difference and (**b**) deviation angle. Due to the 1.4 × SNR gain, Wideband results have lower HWHM in both histograms, indicating better reproducibility. The half width at half maximum (HWHM) of whole brain FA difference distribution dropped from 0.072 to 0.041(56.9%), and the HWHM of fiber deviation angle histogram decreased from 25.3° to 16.2° (64%).

**Figure 10:**
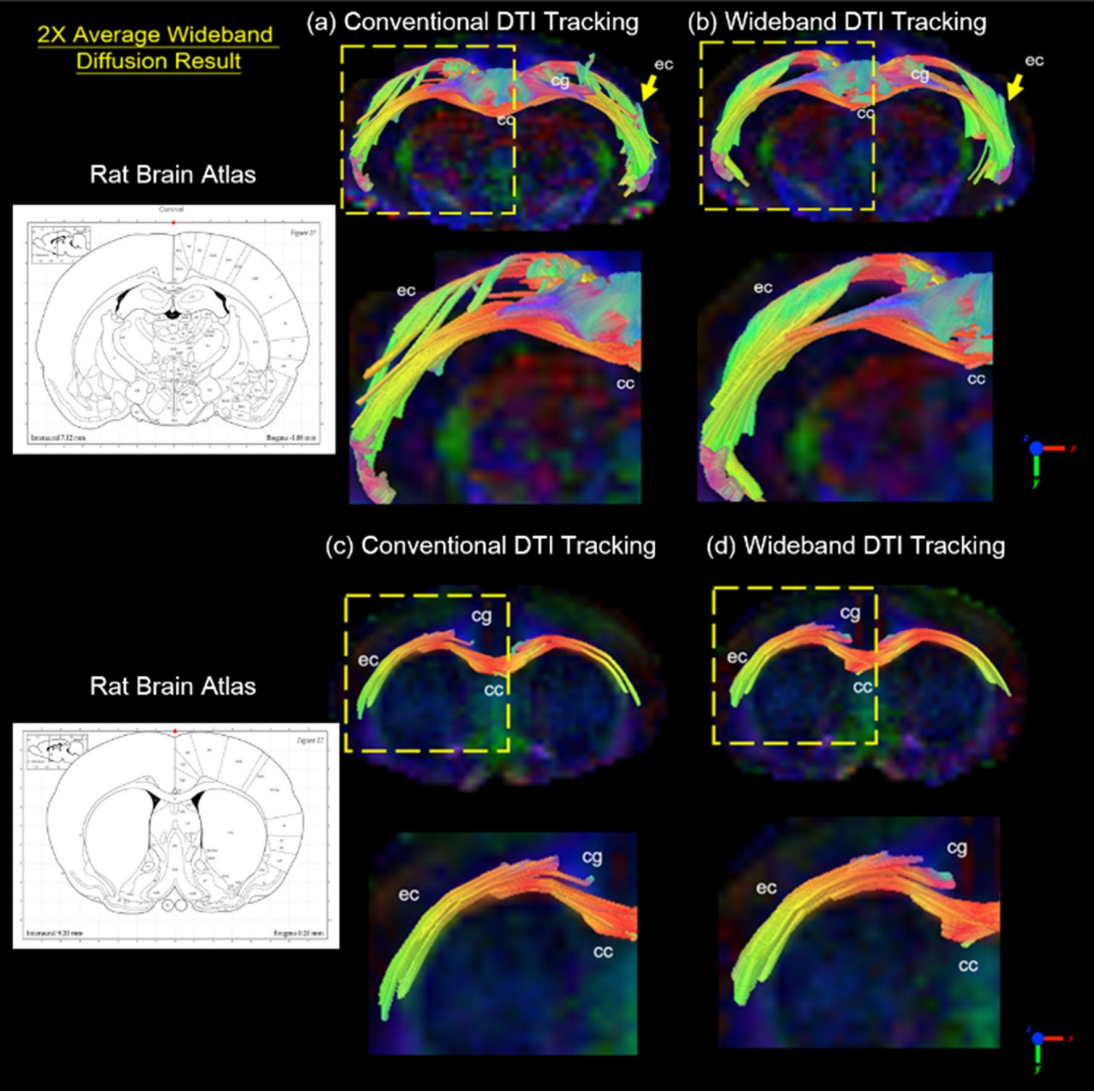
Diffusion Tracking of 2X-Average Coherent Wideband EPI. Images are magnified for the comparison between conventional EPI (SNR = 76.1) and SNR enhanced Wideband EPI (SNR = 105.2) tractography (2 × scan numbers; same scan time). The Wideband result shows higher SNR and uniform tracking in the external capsule (ec), corpus callosum (cc), and cingulum (cg).

**Figure 11:**
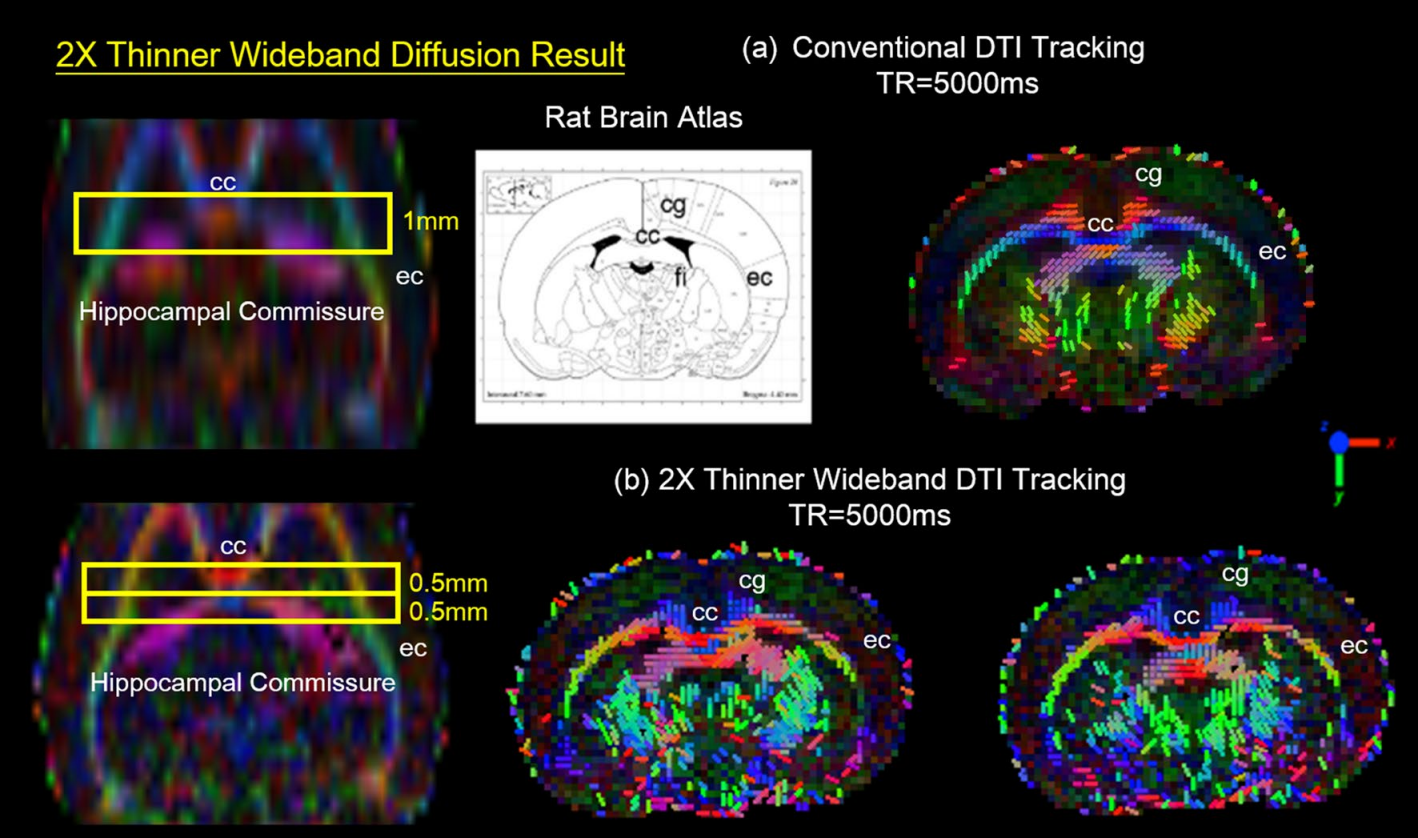
The diffusion tracking of 2X Thinner Wideband results. Conventional EPI DTI images (SNR = 55) and thinner Wideband EPI DTI images (2 × thinner slices; same scan time). The conventional low SNR DTI scan yields specious tracking results, particularly in the corpus callosum (cc), and cingulum (cg). The comparison shows that the Wideband result presents more details of neuro-architecture and consistent tracking in fimbria (fi), ec, cc, and cingulum (cg).

**Figure 12:**
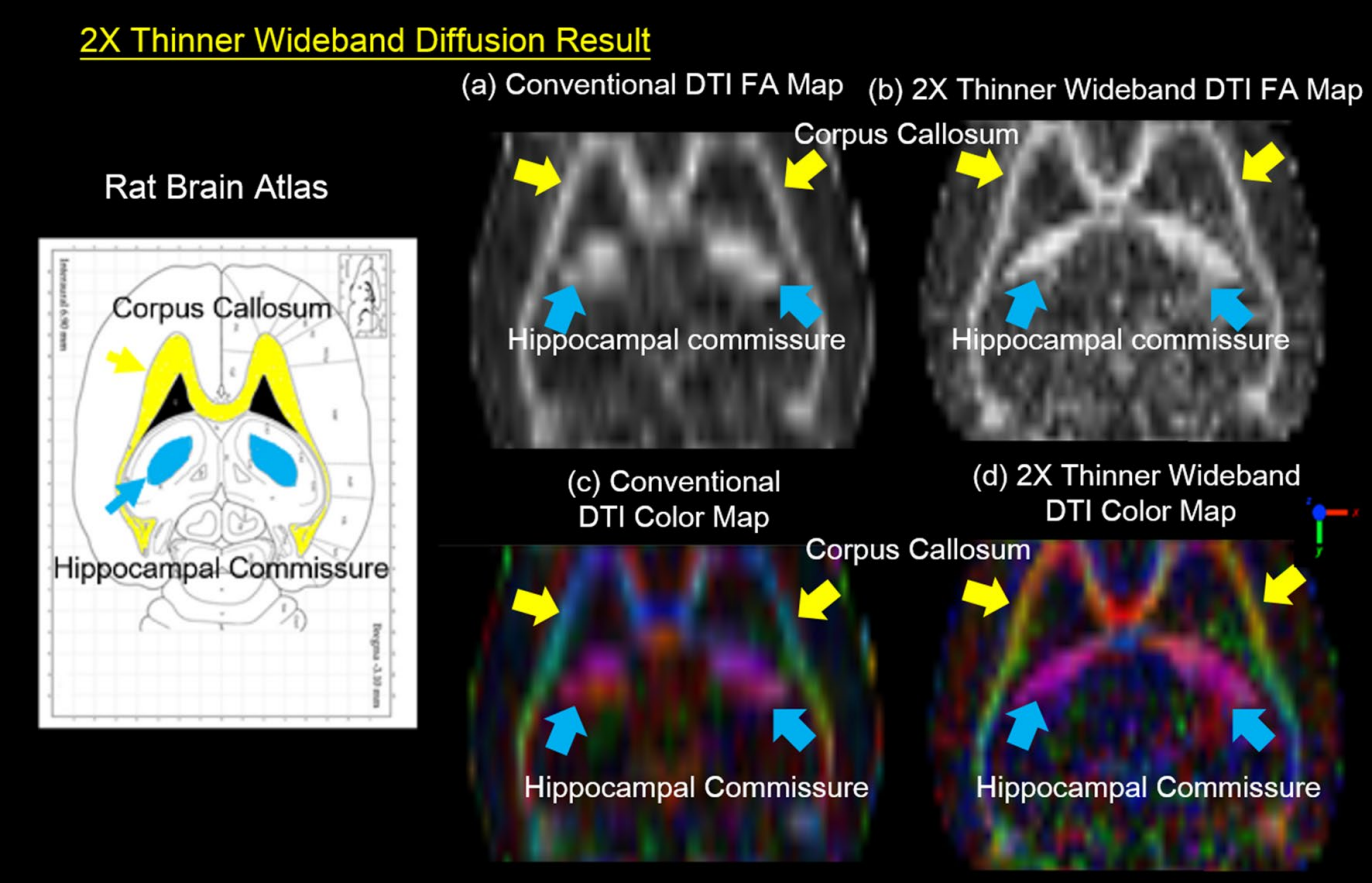
The 2 × thinner wideband diffusion study. DTI FA maps (**a** and **b**) and DTI Color maps (**c** and **d**) are magnified to compare the conventional and thin-slice Wideband results. Since the partial volume effect is reduced, the precision of quantitative DTI analysis is the uniformity of tracking are enhanced in the Hippocampal Commissure (HC) and corpus callosum (cc).

Figure 7 presents high temporal resolution DTI results achieved by the Coherent Wideband spin-echo EPI technique. Under the same scan time, the time-saving effect can also be traded off against SNR improvement by increasing the number of averages, as demonstrated in Figs. 8, 9 and 10. To reduce the partial volume effect of DTI tracking in fiber kissing and fiber crossing, the slices can also be thinned by half as shown in Figs. 11 and 12.

#### 2X-average number coherent wideband EPI DTI

In this experiment, a 1.4X SNR enhancement for 2X average Wideband images with the same acquisition time was observed as expected in Fig. 8. The comparison shows a slight FA value rise with a clear standard deviation decrease for 2-average Wideband enhanced DTI. To quantify the accuracy of diffusion MRI in axonal fiber orientation, each DTI scan was performed twice, and the fractional anisotropy (FA) difference and deviation angles (DA) for repeated scans were determined.

As Fig. 8 demonstrates, 2X-Average Wideband DTI, resulting in higher SNR, the half width at half maximum (HWHM) of whole brain FA difference distribution dropped from 0.072 to 0.041, and the HWHM of fiber deviation angle histogram decreased from 25.3° to 16.2°. Both plots indicate that the reproducibility of FA and fiber angle improved along with the 1.4X SNR gain in Fig. 9.

The conventional DTI tractography images and the SNR-enhanced Wideband DTI images are shown in Fig. 10. With higher SNR, the Wideband DTI tractography shows a more robust tracking result in the external capsule (ec), corpus callosum (cc), and cingulum (cg) without noticeable erroneous forks.

#### 2X thinner slice coherent wideband EPI DTI

A low SNR diffusion tractography is susceptible to the partial volume effect which deteriorates analysis results. In this experiment, the SNR for DTI results was set to 55, a value lower than previous DTI studies, to demonstrate the partial volume effect for DTI analysis. Under these circumstances, a conventional DTI scan yields specious tracking results, particularly in the cc and cg regions. On contrary, the 2 × thinner Coherent Wideband DTI tractography with 0.5 mm thickness (Fig. 11b) presents more detailed neuro-architecture and more accurate tracking results according to the atlas. As Fig. 11 demonstrates, the demarcation of the axonal fiber orientation is also improved with thinner slices.

Figure 12 is the coronal view of its DTI results, presenting the entire CC fiber tract. The FA maps illustrate the diffusivity, and the color maps represent the orientation of axons. As shown in the FA maps (Fig. 12a and b), the 2X-Thinner Wideband DTI result shows accurate fiber continuity, especially for the Hippocampal Commissure (HC) region. The consistency of fiber tracking for the CC region is also improved, as demonstrated in the color maps (Fig. 12c and d).

### Resting result

Since current resting fMRI analyses rely on regional locality, the accuracy of results highly depends on its temporal signal to noise ratio (tSNR)^35^, which means that more time points and higher temporal resolution can both improve the precision and stability of fMRI results. In the application results of resting fMRI, the Coherent Wideband gradient-echo EPI technique doubled the sampling rate to acquire more data points under the same scan time for better resting-fMRI analysis. (Parameter as shown in Table.1) For Wideband MRI with N = 240, every two repetitions are averaged to one in tSNR analysis.

**Table 1:** Resting-fMRI scan parameter.

Parameter	FOV (cm^2^)	Matrix	TR/TE (ms)	Repetition and Dummy-Scan	Scan Time
Conventional Scan	2.5 × 2.5	64 × 64	2000/25	180* 60	6’
Coherent Wideband Scan	2.5 × 5	64 × 128	1000/25	360^†^ 120	6’
Coherent Wideband Scan	2.5 × 5	64 × 128	1000/25	180* 60	3’

*Among the 180 points, the first 60 are dummy scans and are excluded in the analysis (effective repetition = 120).

^†^Among the 360 points, the first 120 are dummy scans and are excluded in the analysis (effective repetition = 240).

**Figure 13:**
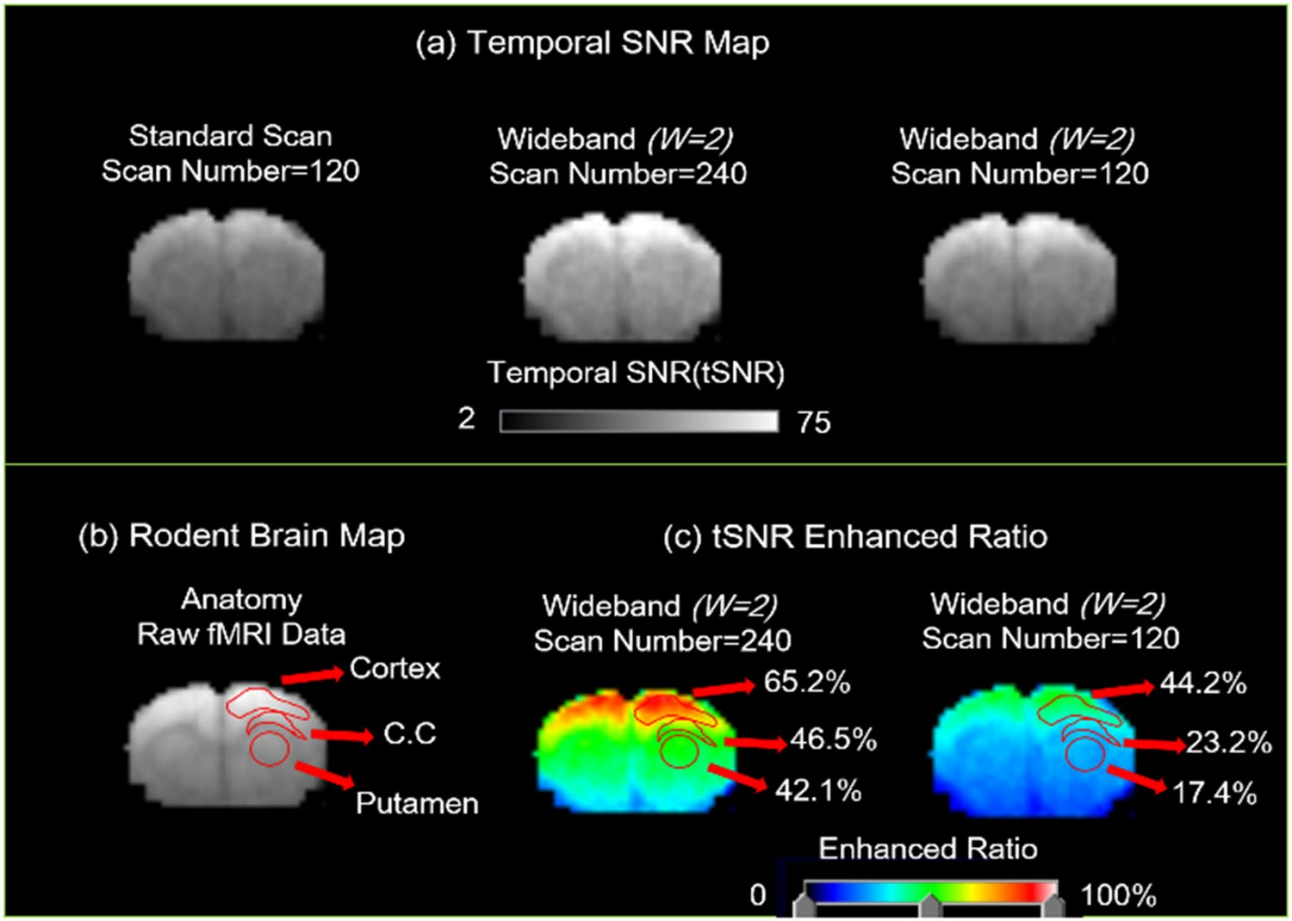
Temporal signal to noise ratio map. In the resting functional MRI experiment, the tSNR map was boosted by 65.2% to 70.9 ± 9.22 by doubling the scan number, and was elevated from 43.28 ± 9.65 to 62.41 ± 9.52 (44.2%) by shorten the sampling interval in the cortex area. In the corpus callosum and Putamen, by doubling the scan number in the cortex area, tSNR increased by 46.5% and 42.1%; by shortening the sampling interval, tSNR increased from 34.23 ± 11.28 and 32.12 ± 13.22 to 42.17 ± 9.31 (23.2%) and 37.71 ± 9.28 (17.4%).

**Figure 14:**
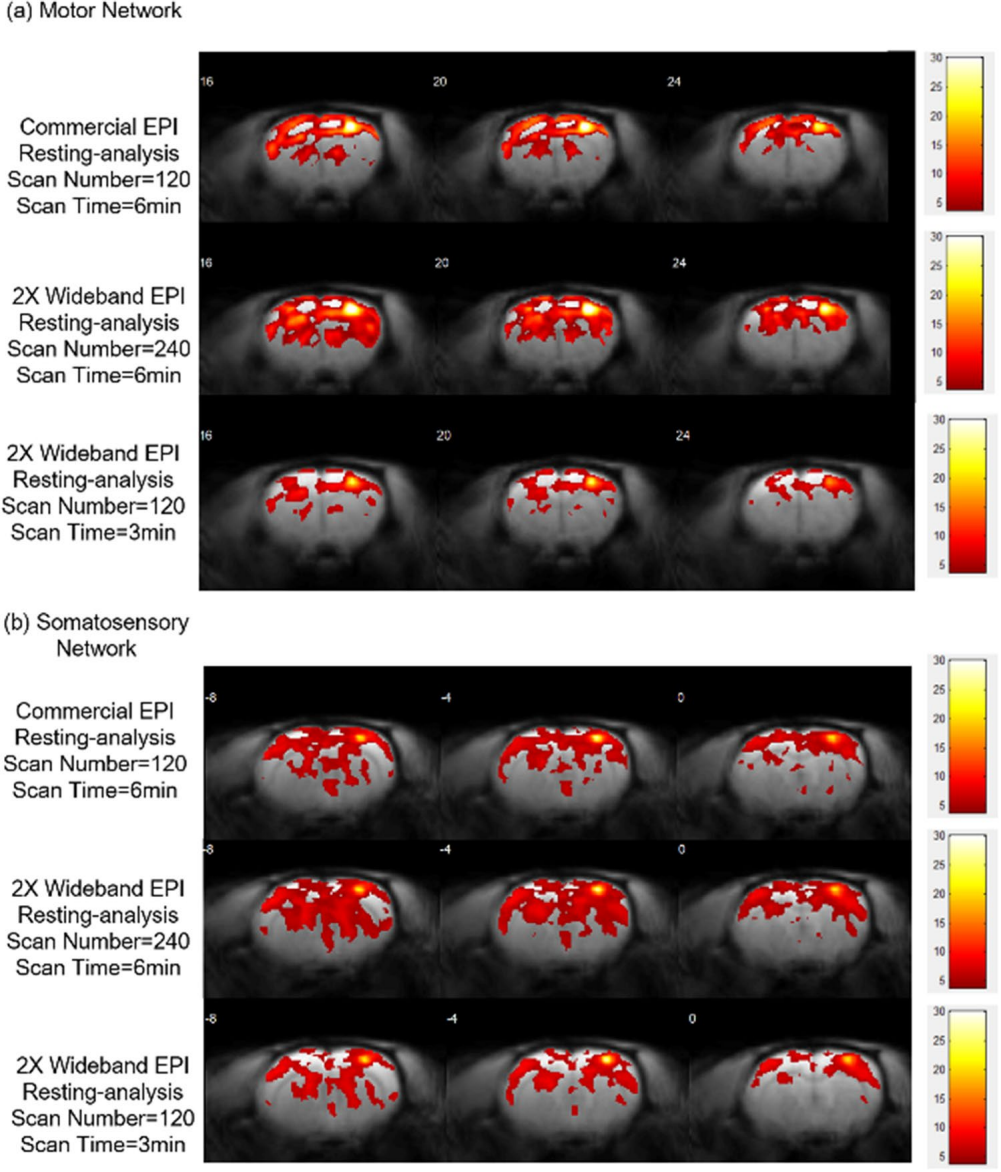
Resting-state functional connectivity. Resting-state functional connectivity with bandpass filtered at 0.01 and 1 Hz. Group resting-state functional connectivity maps (p < 0.005, N = 6) using seed regions in the (**a**) right motor network, (**b**) right somatosensory network. The 2X Wideband EPI (Scan Number = 240) shows increased (1.38 times as conventional scan) regional connectivity, but 2X Wideband EPI (Scan Number = 120) reveals less (0.78 times as conventional scan) connections in the right motor network. In the right somatosensory network, 2X Wideband EPI (Scan Number = 240) presents 1.34 times relative regions but 0.82 times regions are observed in 2X Wideband EPI (Scan Number = 120).

The tSNR of Wideband resting-fMRI is enhanced in Cortex, CC, and Putamen (Fig. 13). The tSNR was elevated by 44.2% from 43.28 ± 9.65 to 62.41 ± 9.52 by shortening the sampling interval and was boosted by 65.2% to 70.9 ± 9.22 by doubling the scan number in the cortex area. This enhancement results directly from the reduction of thermal noise with high sampling point number and the reduction of physiological noise with high sampling rate while using Coherent Wideband EPI. As the experimental result demonstrates, the Coherent Wideband EPI result shows a 38.3% (Fig. 14a and b) increase of associated activation region size in comparison with the traditional resting fMRI result due to the tSNR enhancement in the double-sampling case.

## Discussion

**Figure 15:**
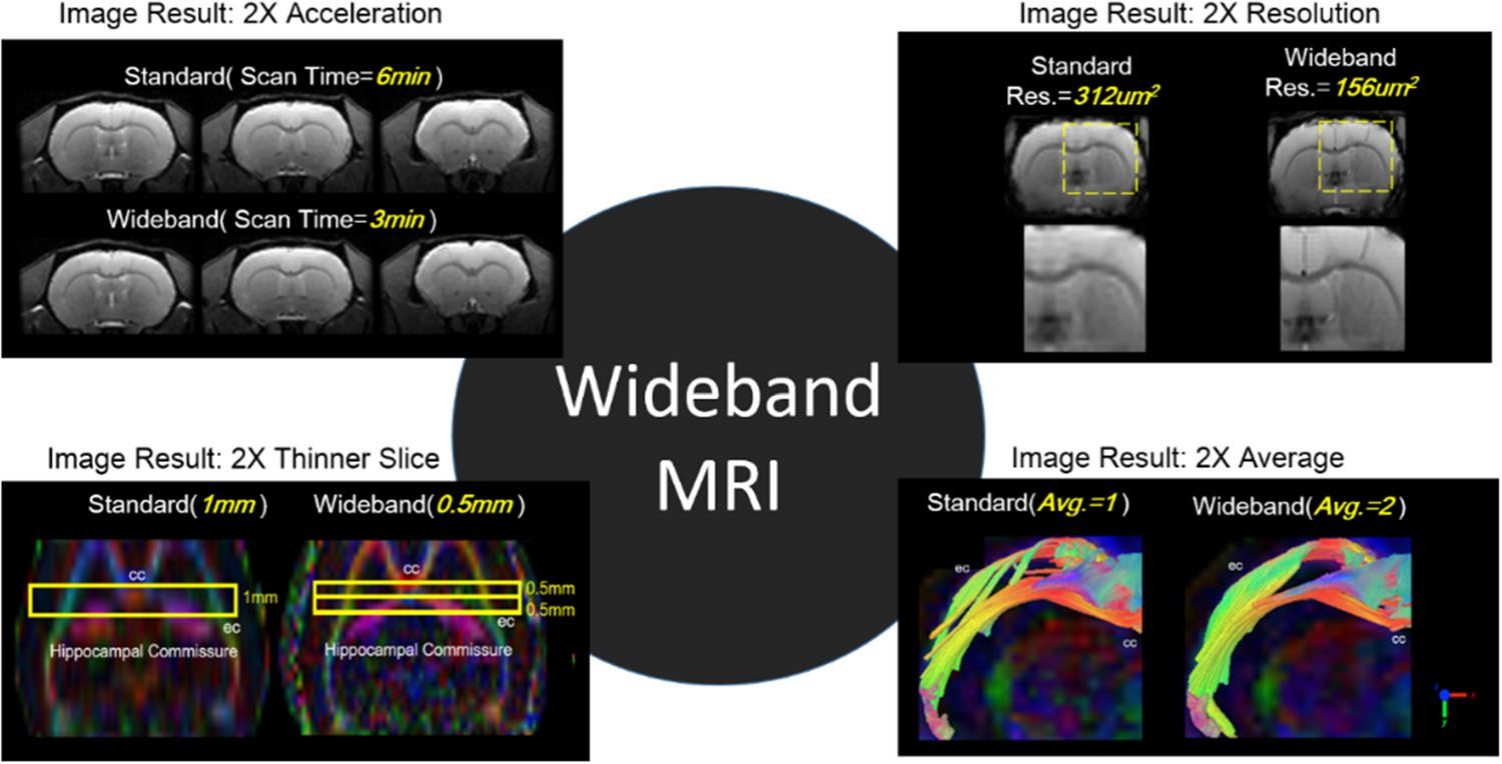
Multiple applications of the Wideband MRI. This study demonstrated multiple benefits of the Coherent Wideband EPI technique, including temporal/spatial resolution enhancement, SNR improvement, and accuracy study.

This study presented multiple applications of the Coherent Wideband EPI technique and demonstrated its multifaceted benefits (Fig. 15). For temporal resolution enhancement, Coherent Wideband EPI scans multiple slices simultaneously to reduce TR and thus total duration, providing results with double speed while the quality remains identical with the conventional results. This method can also obtain double data points under the same scan time to elevate the spatial resolution of rodent brain results. Regarding diffusion applications, under the same scan time, Coherent Wideband EPI can either decrease slice thickness to diminish the partial volume effect of DTI tractography or increase the number of averages to reduce FA difference and fiber deviation angle. As for resting fMRI, the proposed technique doubled the temporal resolution for a significant improvement in analysis results. Nonetheless, the Coherent Wideband EPI technique still has room for improvement in multiple aspects, which we’ll respectively elaborate on in the following sections.

### TE limitations

To achieve a W-fold acceleration with Wideband, the imaging process acquires W times as many points as the conventional scan. These additional data points prolong the TE of Wideband scans and exacerbate T2 decay, which could be more severe for the high field. To resolve this issue, the receiver bandwidth for Coherent Wideband EPI should be also increased to fully restore signal strength. Hence, the gradient specifications of MRI systems, including slew rate and gradient amplitude, need to be compatible with wider bandwidth for the highest possible W for Coherent Wideband. For the current Bruker 7 T Biospec animal system, the maximum achievable W is 3. To achieve further acceleration rate solely with the Coherent Wideband technique, a gradient upgrade is required.

Nevertheless, the Coherent Wideband EPI technique has high compatibility with other acceleration methods, including Hadamard encoding and parallel imaging techniques. Therefore, further acceleration is achievable without further increasing TE or bandwidth to avoid hardware limitations.

### TR/T1 effect in wideband experiments

The proposed Coherent Wideband technique achieves a W-fold acceleration by reducing TR to 1/W of the original TR of a standard scan. Since shortened TR reduces SNR due to the T1 effect, the Wideband technique acquires W times as many points as the standard scan to provide a $\sqrt{W}$-fold SNR boost. This process can ensure Wideband results to have comparable SNR as standard results if a sufficiently long TR value is chosen, as the experimental results in this study demonstrate. However, due to the T1 effect, this process won’t be as effective for shorter TR values.

**Figure 16:**
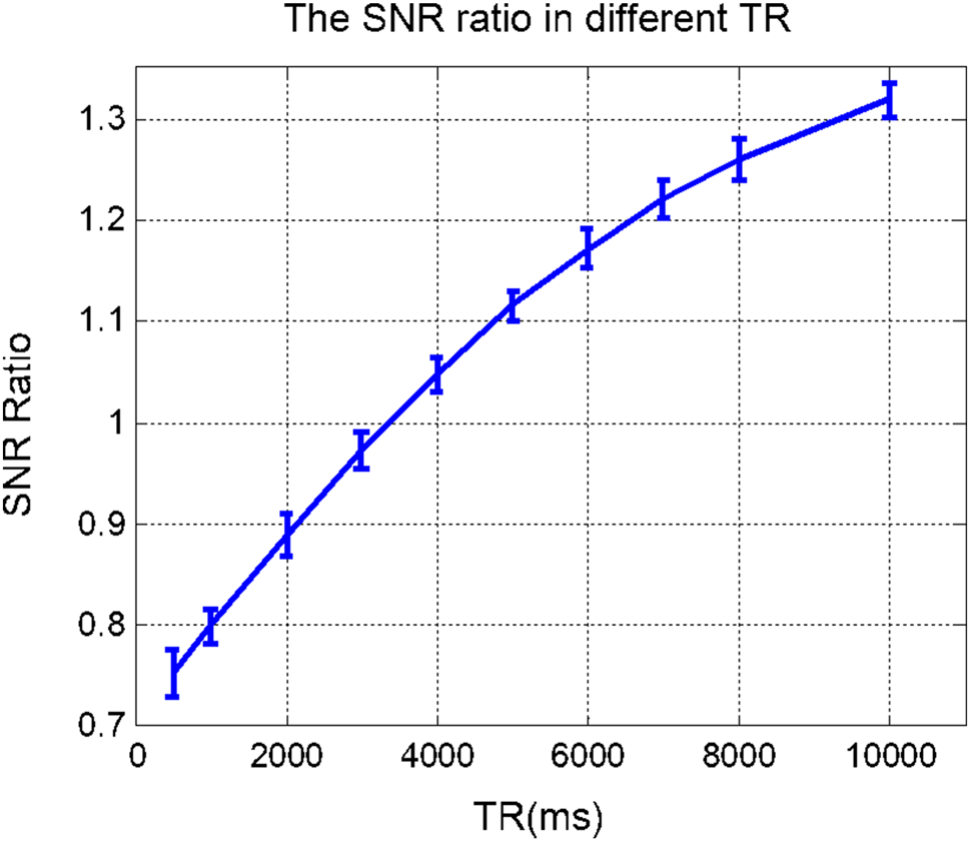
SNR difference between Wideband and standard scans. Shortened TR reduces SNR due to the T1 effect, the Wideband technique acquires W times as many points as the standard scan to provide a $\sqrt{W}$-fold SNR boost. As the result, the SNR difference between Wideband and standard scans depends on the chosen TR values.

To further investigate this phenomenon, the following experiment was conducted to measure and compare the SNR of rat brain images for a standard scan and a Wideband scan when different TR values were chosen. The SNR difference between standard and Wideband scans is depicted as the SNR ratio in the following chart/plot. (As shown in Fig. 16).

In 7 T environments, T1 is approximately 1900 ms for the gray matter of rat brains. As the results demonstrate, for TR between 3000 and 5000 ms, the Wideband scan and standard scan provide similar SNR values, which is consistent with our rat brain experiments in this study. In contrast, for TR shorter than 3000 ms, the SNR for Wideband scan decreases. In general, for commonly used TR values in rat brain experiments, Wideband MRI provides acceleration benefits and comparable SNR with conventional techniques.

### The separation limitation of multi-slice distance

**Figure 17:**
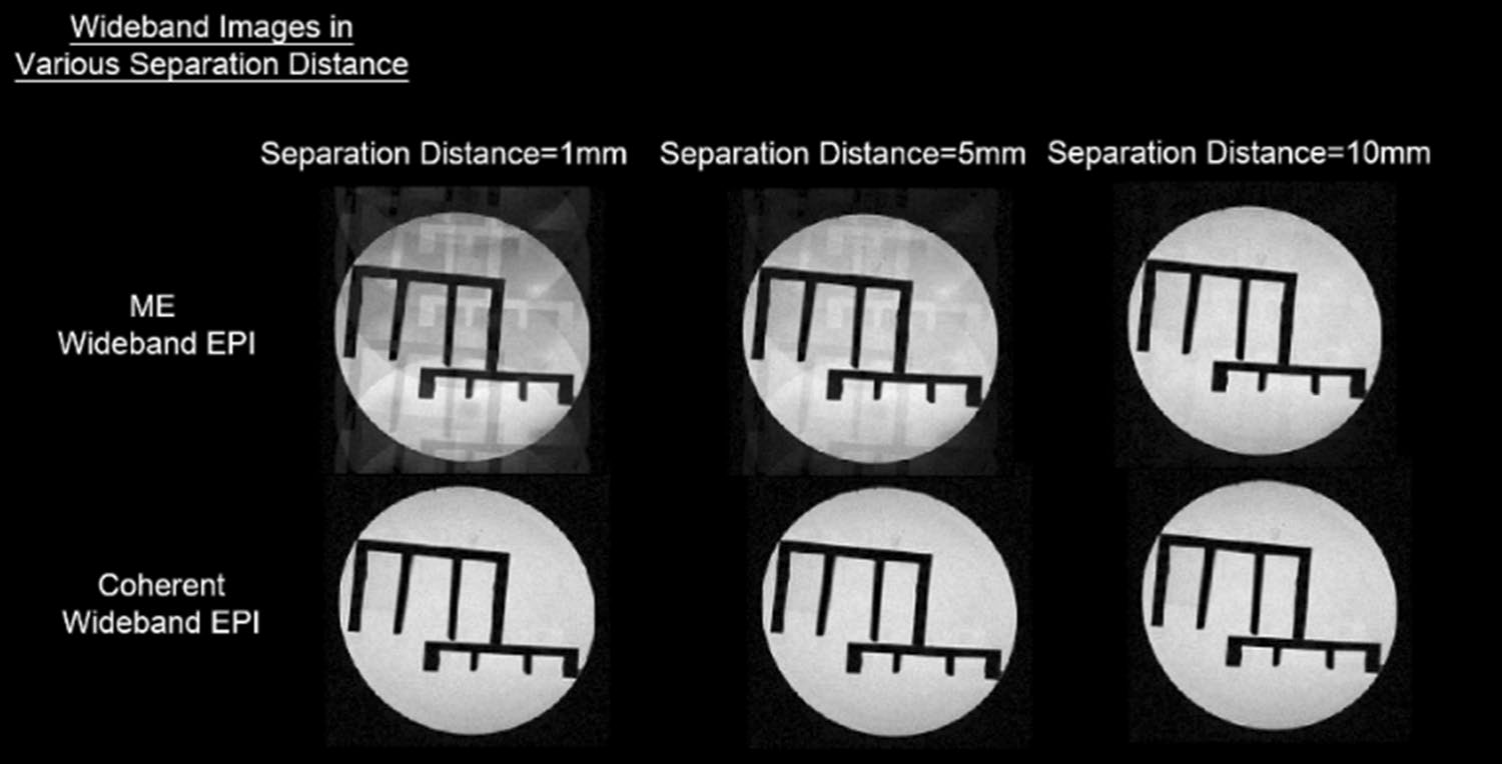
Slice distance effect for ME-Wideband and Coherent Wideband EPI. Since ME-Wideband applies stronger refocusing gradients for image separation, its image quality would be limited by the slice distance. As slice distance decreases, ME-Wideband images deteriorate with severe artifacts while Coherent Wideband images maintain consistent quality.

The magnitude of separation gradient depends on the distance between two simultaneously-excited slices – proximate slices require stronger gradients for successful separation on the image domain. Since the gradient slew rate is limited, the designed amplitude for additional gradients couldn’t be reached for adjacent slices. This results in incorrect phase for acquired signals which shifts the images to undesignated locations and forms artifacts. As shown in Fig. 17, since ME-Wideband (Fig. 1) applies 3 times stronger refocusing gradient to offset the accumulated phase, apparent artifacts appeared and became more obvious as slice distance decreased to 1 mm. On the other hand, the Coherent Wideband EPI method utilizes bipolar separation gradients to keep isochromatic spins in phase, which massively reduces the required separation gradient strength. Hence, proximate simultaneously-excited slices can still be acquired and maintained consistent quality.

### DTI precision analyses

The trade-off between sufficient SNR and image resolution is often a dilemma for MRI researchers. Toward this end, this study presented two usages of Wideband EPI, thin-slice and high-SNR Coherent Wideband DTI, to enhance the calculated MRI image quality and meet the demands for precision medicine studies.

**Figure 18:**
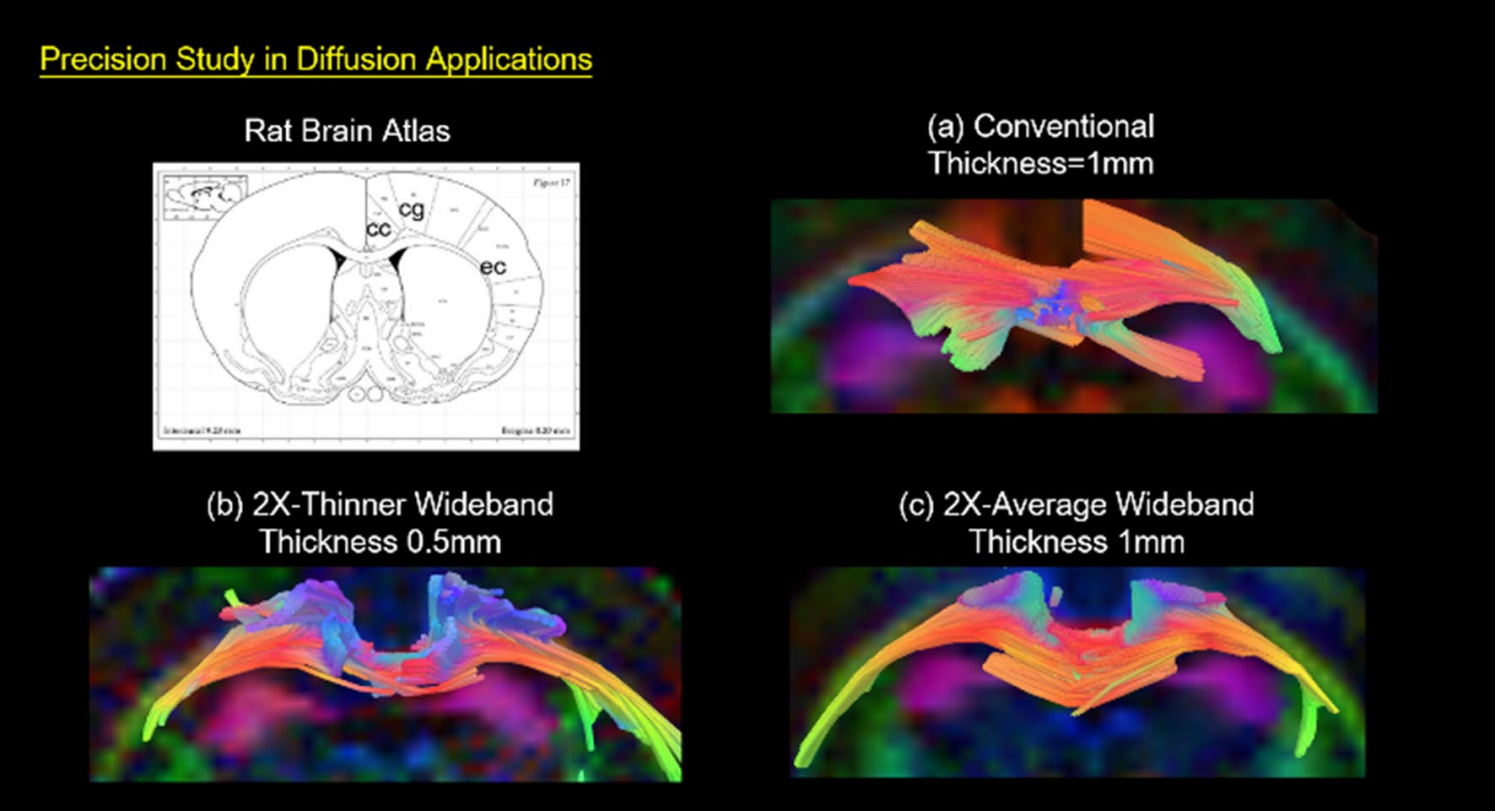
Tracking results of conventional and Wideband DTI. The tractographies of conventional EPI, 2X-Thinner Wideband and 2X-Average SNR enhanced Wideband EPI. The SNR of conventional DTI result is 55, slightly lower than previous DTI studies in Fig. 7. In comparison, the 2X-Thinner Wideband result (**b**) shows better delineation for neuro-architectures, and 2X-Average Wideband result (**c**) reveals consistent tracking in the corpus callosum (cc) and cingulum (cg) regions.

In DTI applications, the partial volume effect causes specious tractography due to fiber kissing or crossing^36^, especially in low-SNR circumstances. As demonstrated in our DTI study results (Fig. 18b), 2X-Thinner Wideband DTI provided robust tracking results, with distinct lateral tracks in the CC region and anteroposterior tracks in the CG region. 2X-Average Wideband DTI also provides smoother tracking results with better reproducibility due to its 1.4x-SNR enhancement (Fig. 18c). On contrary, in the conventional DTI, complex fiber orientations cannot be distinguished with lower spatial resolution, therefore only the dominant lateral tracks remained in the tractography along with some erroneous anteroposterior forks (Fig. 18a). This comparison highlights the importance of Wideband: when the SNR for a DTI scan isn’t sufficient to provide a reasonable tractography, Wideband can either enhance SNR or reduce slice thickness to elevate the accuracy of neuro-tractography analyses.

### The effect of increasing field of view

Increasing FOV/readout will cause blurring in the y-direction since the high-frequency signal attenuates. Due to the limitation of y-direction time resolution, the longer readout will lead to more serious distortion. Nonetheless, these distortions can also be effectively solved by built-in field map and pre-emphasis for the increased FOV in MRI scanners.

**Figure 19:**
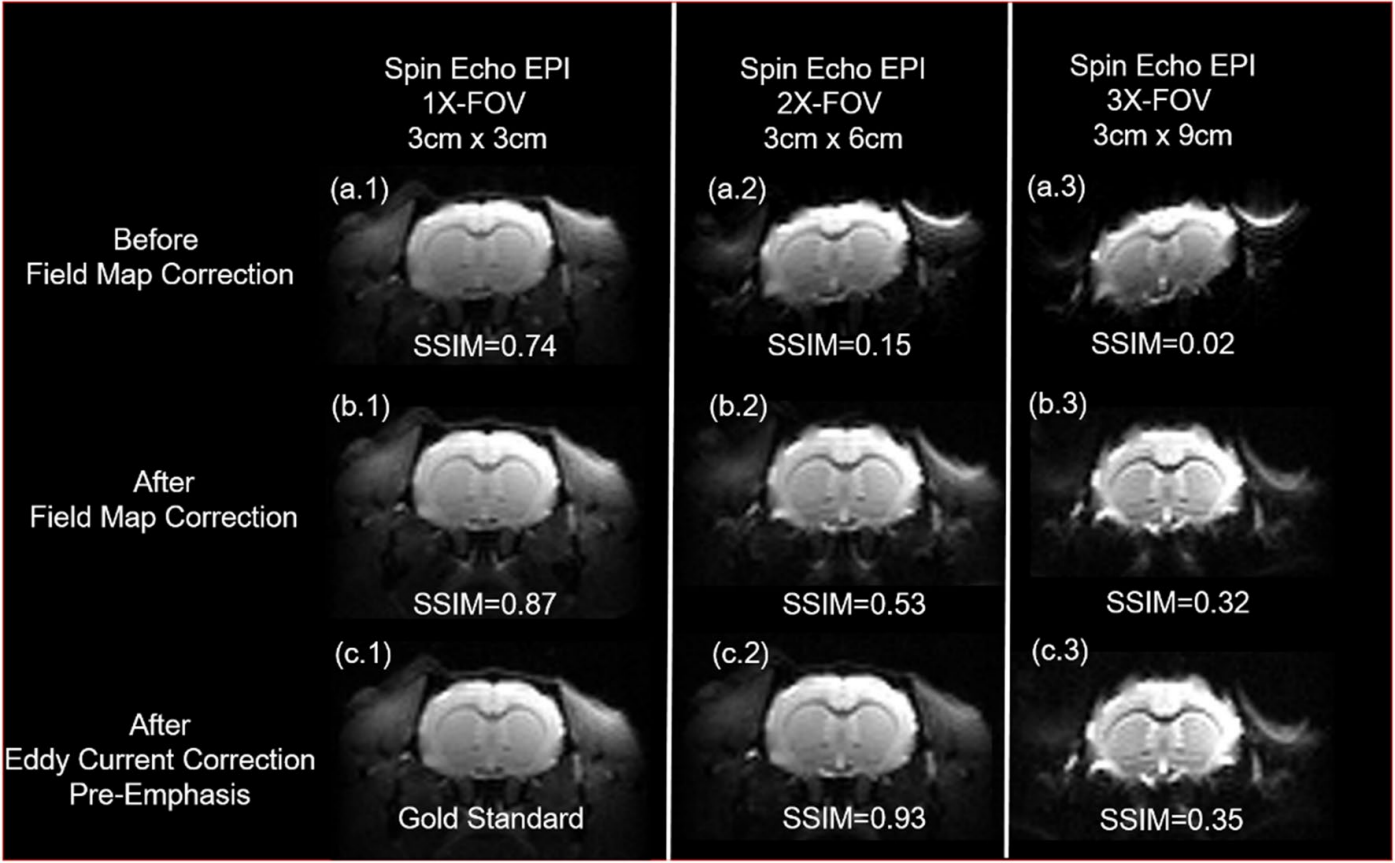
Spin echo EPI images of different field of view. The rat brain images of conventional EPI, 1X-FOV, 2X-FOV and 3X-FOV scans. This figure demonstrates how extended FOV (y-direction) intensifies distortion and blur, and how they can be effectively corrected with the built-in field map and manually-adjusted pre-emphasis processes on the Bruker system. All SSIM are calculated with (**c.1**) as the gold standard.

The comparison of the standard (as shown in Fig. 19a.1, b.1, c.1) and enlarged FOV images in the figure below clearly demonstrated how these correction methods effectively reduce distortion. As demonstrated, for 2X-FOV (as shown in Fig. 19a.2, b.2, c.2), which is the case of this study, the distortions can be effectively solved by built-in field map and pre-emphasis (SSIM = 0.93). However, for 3X-FOV (Fig. 19a.3, b.3, c.3), these two methods failed due to more severe distortion in the longer readout. Therefore, better distortion correction combined with other acceleration technologies are required for 3X-FOV (Fig. 19c.3).

### Compatibility with current acceleration techniques

The Coherent Wideband EPI technique is highly compatible with multiple existing MRI acceleration techniques, such as Hadamard encoding, and parallel imaging. The Hadamard encoding method adjusts RF phase to reconstruct simultaneously-excited slices, so even more slices can be simultaneously acquired with its combination with Wideband. With multi-coil arrays, Coherent Wideband can also combine with other SMS techniques to reconstruct images with coil sensitivity information and utilize parallel imaging techniques like SENSE and GRAPPA to achieve further acceleration.

### Diffusion tractography between conventional and wideband scan

**Figure 20:**
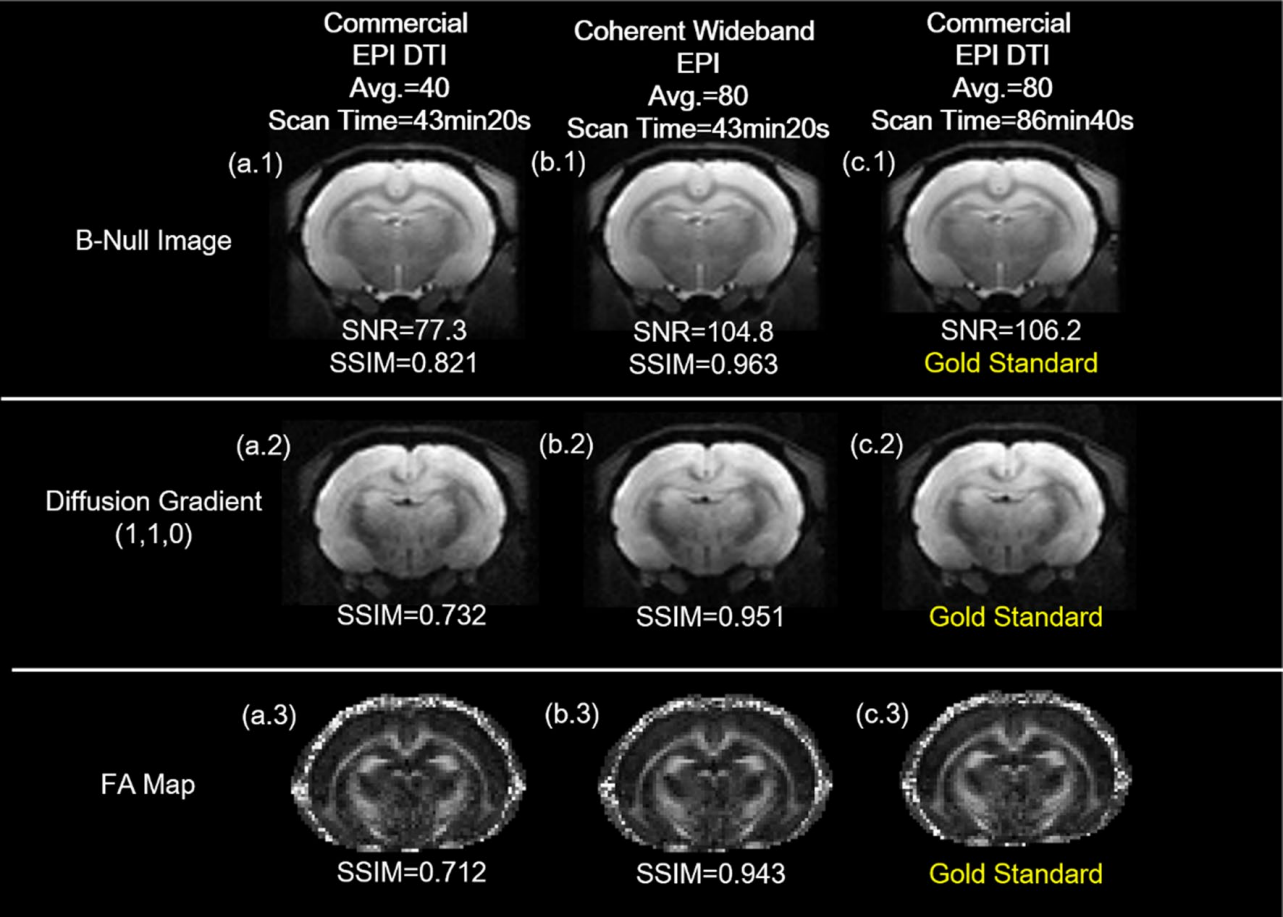
The image quality between conventional and wideband scan. The SSIM for Wideband EPI is 0.963 for B-Null, 0.951for Diffusion Gradient, and 0.943 for FA Map. Since the SSIM for two repeated gold standard scans is around 0.95–0.96, this indicates that the Wideband result is highly consistent with the gold standard.

We perform another DTI experiment using conventional EPI with double repetition as the gold standard (Fig. 20c.1, c.2, c.3). To quantify the accuracy of diffusion MRI in axonal fiber orientation, each DTI scan was performed twice and the FA difference for the whole brain was determined. To determine structural similarity, SSIM is calculated by comparing images with the corresponding gold standard.

In general, the SSIM for two repetitions of conventional EPI scans is around 0.95–0.96. As the figure demonstrates, the SSIM for Wideband EPI is 0.963 for B-Null (Fig. 20b.1), 0.951 for Diffusion Gradient (Fig. 20b.2), and 0.943 for FA Map (Fig. 20b.3). The conventional scan with less average number shows the lower SNR value (Fig. 20a.1, a.2, a.3). This suggests that Coherent Wideband EPI provides results that are highly consistent with the gold standard in only half of the original scan time.

**Figure 21:**
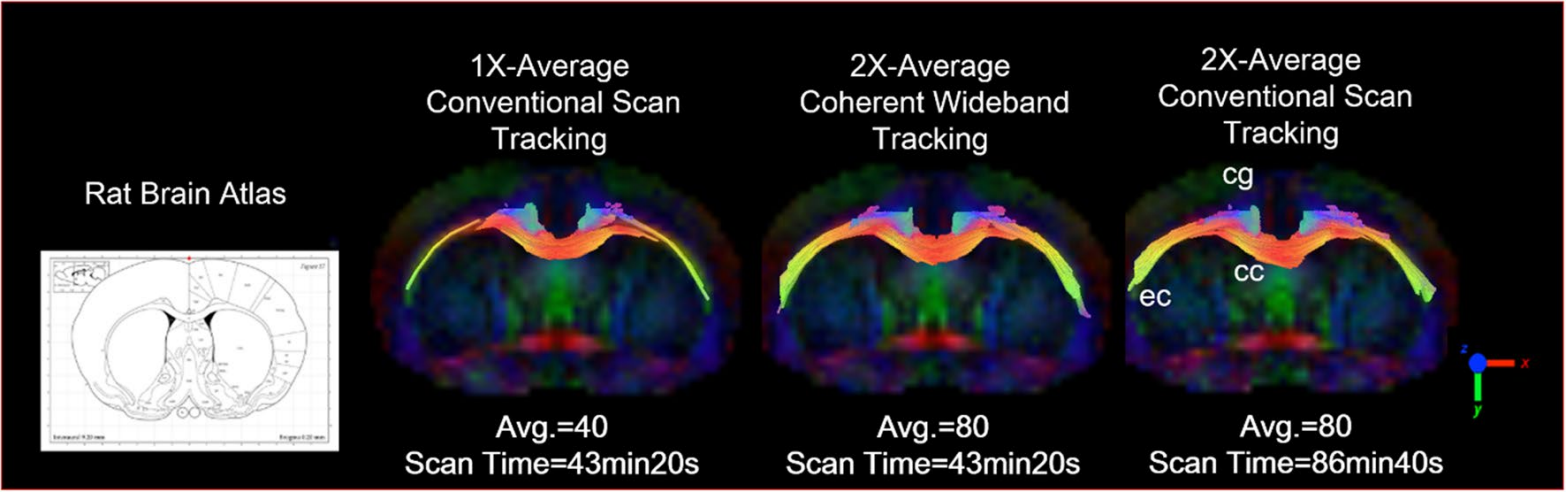
Diffusion tractography between conventional and wideband scan. In comparison with 1X-average conventional EPI, the Wideband result shows higher SNR and tracking results closer to the gold standard (2X-Average Conventional Tracking) in the external capsule (ec), corpus callosum (cc), and cingulum (cg).

For DTI tractography, a set of conventional EPI result with 2X-Average is attached as the gold standard for the comparison between conventional EPI and SNR-enhanced Wideband EPI. The images are all magnified for better clarity (Fig. 21).

In conclusion, we successfully validated our Wideband DTI result using the gold standard. The SSIM values, DTI histogram, and tractography all show that Coherent Wideband EPI provides results that are highly consistent with the gold standard in only half of the original scan time.

## Conclusion

This study proposed the Coherent Wideband EPI technique and demonstrated its multifaceted benefits without the usage of phase array coils. This technique also adopted a precise phase alignment method to minimize the N/2 ghost artifacts in conventional EPI images, reducing the ghost factor from 6.3 to 3.1%.

The Coherent Wideband EPI technique was employed on a pre-clinical animal system for various applications to demonstrate its versatility. First, a phantom experiment was conducted to verify the consistency between conventional EPI and Wideband EPI images. Subsequently, the structural images for rodent brains with double imaging speed and double spatial resolution were successfully acquired to illustrate how Wideband elevates MRI efficiency. For DTI, under the same scan time, Coherent Wideband EPI provided more accurate and consistent tracking results by either increasing the z-resolution or improving image SNR. Quantitative diffusion analysis (HWHM of FA difference and DA) of the SNR-enhanced Wideband provided the lower fluctuations (FA difference: 56.9%, DA: 64%) for outcome compared to conventional scans.

Regarding the resting fMRI study, the tSNR map was elevated by 44.2% from 43.28 ± 9.65 to 62.41 ± 9.52 by shortening the sampling interval and was boosted by 65.2% to 70.9 ± 9.22 by doubling the scan number in the cortex area. This ends up as a 38.3% increase of associated activation regions in comparison with traditional resting fMRI results due to the tSNR enhancement in the double-sampling case.

In conclusion, the proposed Coherent Wideband EPI technique provides a novel and reliable alternative to achieve simultaneous multi-slice imaging without phase array coils, which could be especially advantageous for small animal pre-clinical MRI scanners. The comprehensive enhancements of this technique not only benefit neuro-architecture imaging, but also advance DTI and fMRI connectivity analysis studies which emphasizes the significance of precise quantitative analyses. Since the thermal noise of MR images may exacerbate error propagation with each step of the DTI/fMRI neuro-estimations, the Wideband technique, which provides credible anatomical information/precision analysis, can greatly contribute to the biological plausibility of neuro-informatics. In summary, Coherent Wideband EPI will provide faster, higher resolution, thinner slice imaging, or higher signal-to-noise MR imaging for precision neuro-architecture studies to facilitate future medical researches.

## Data Availability

The image data generated and presented in this study are available from the corresponding author on reasonable request.
